# Paved Roads as Ecohydrological Modifiers: Slope‐Driven Runoff Is Associated With Roadside Establishment of the Atlantic Halophyte *Cochlearia danica* in Continental Europe

**DOI:** 10.1002/ece3.74212

**Published:** 2026-09-01

**Authors:** Szabolcs Kis, Jenő Nagy, Attila Molnár V.

**Affiliations:** ^1^ HUN‐REN–UD Conservation Biology Research Group, Department of Botany, Faculty of Sciences and Technology University of Debrecen Debrecen Hungary; ^2^ Department of Pharmacognosy, Faculty of Pharmacy University of Debrecen Debrecen Hungary; ^3^ Juhász‐Nagy Pál Doctoral School University of Debrecen Debrecen Hungary

**Keywords:** anthropogenic linear habitats, continental range expansion, halophytes, roadside vegetation, road‐surface runoff, species distribution modelling

## Abstract

Linear transport infrastructures are widely recognised as corridors for plant dispersal; however, their role in modifying local abiotic conditions remains less explored. In this study, we examined whether slope‐driven, unidirectional precipitation runoff from paved roads is associated with differences in roadside soil‐moisture and local abundance of *Cochlearia danica* , an Atlantic halophytic species currently spreading into continental Europe. Targeted roadside surveys were conducted in Slovakia, Hungary, Serbia and Romania, complemented by comparative soil‐moisture assessments on opposing sides of sloping road sections. Downslope verges receiving concentrated runoff consistently showed higher gravimetric soil‐moisture and were associated with larger *C. danica* populations than upslope verges. Population size showed a positive association with local variation in soil‐moisture. Supplementary species distribution modelling based on bioclimatic variables predicted low macroclimatic suitability for the study region, indicating that several recorded roadside populations occur under marginal conditions. In addition to Hungary, *C. danica* was documented for the first time in Serbia and Romania, with evidence suggesting local establishment of revisited sites. Our results suggest that paved road design may create small‐scale ecohydrological asymmetry along road verges, potentially contributing to the establishment of Atlantic halophytes in continental roadside habitats.

## Graphical abstract

We examined whether slope‐driven runoff from paved roads alters roadside soil‐moisture in ways that facilitate the spread of *Cochlearia danica* , an Atlantic halophyte expanding into continental Europe. Downslope verges receiving concentrated runoff showed higher soil‐moisture and supported larger populations than upslope verges, despite low macroclimatic suitability predicted by species distribution models. These results suggest that road design can locally modify hydrological conditions and facilitate plant invasion along transport corridors.

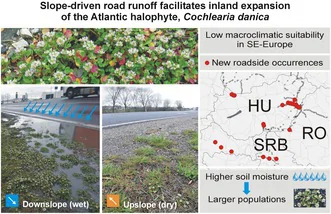

## 1 Introduction

The expansion of transport infrastructure has profoundly reshaped plant dispersal processes across landscapes, with roads and railways acting as effective linear corridors for a wide range of species (Tikka et al. 2001; Medvecká et al. 2018). Vehicle‐mediated seed transport, air turbulence and roadside disturbance are well‐established mechanisms contributing to this phenomenon (Zwaenepoel et al. 2006; von der Lippe et al. 2013; Ansong and Pickering 2013). Consequently, both alien and native pioneer species frequently establish along road verges, often expanding beyond their original biogeographical limits (Šerá 2008; Dítě and Dítětová 2016; Fekete et al. 2021; Molnár, Bak, et al. 2024).

Beyond facilitating dispersal, paved roads can substantially modify adjacent soil characteristics. Road construction and maintenance can alter soil compaction, salinity, electrical conductivity and pH, thereby shaping roadside vegetation patterns (Gelbard and Belnap 2003; Mills et al. 2021). De‐icing practices further intensify these effects by increasing salt inputs into roadside soils (Shannon et al. 2020; Bak et al. 2025). While such chemical and physical alterations have been widely studied, the hydrological consequences of road design, particularly the redistribution of precipitation, have received less attention in plant ecological research.

Among the abiotic effects of roads, hydrological alteration is particularly relevant but remains comparatively less studied in relation to roadside plant establishment (Forman and Alexander 1998; Spellerberg 1998; Coffin 2007). Road networks alter surface water flow, drainage and sediment dynamics, which can have downstream impacts on soil‐moisture regimes and associated plant communities (Forman and Alexander 1998; Spellerberg 1998). Although much of the road ecology literature has focused on structural habitat effects and pollutant transport in runoff (e.g., heavy metals, salts) (Li et al. 2014), research also indicates that road‐induced hydrological changes can affect water availability in adjacent soils and thereby influence vegetation composition and biomass distribution (Coffin 2007; Kastridis 2020). These ecohydrological effects are increasingly recognised as a significant but understudied component of road‐vegetation interactions, especially in habitats where moisture availability is a limiting factor for plant establishment and growth.

**FIGURE 1:**
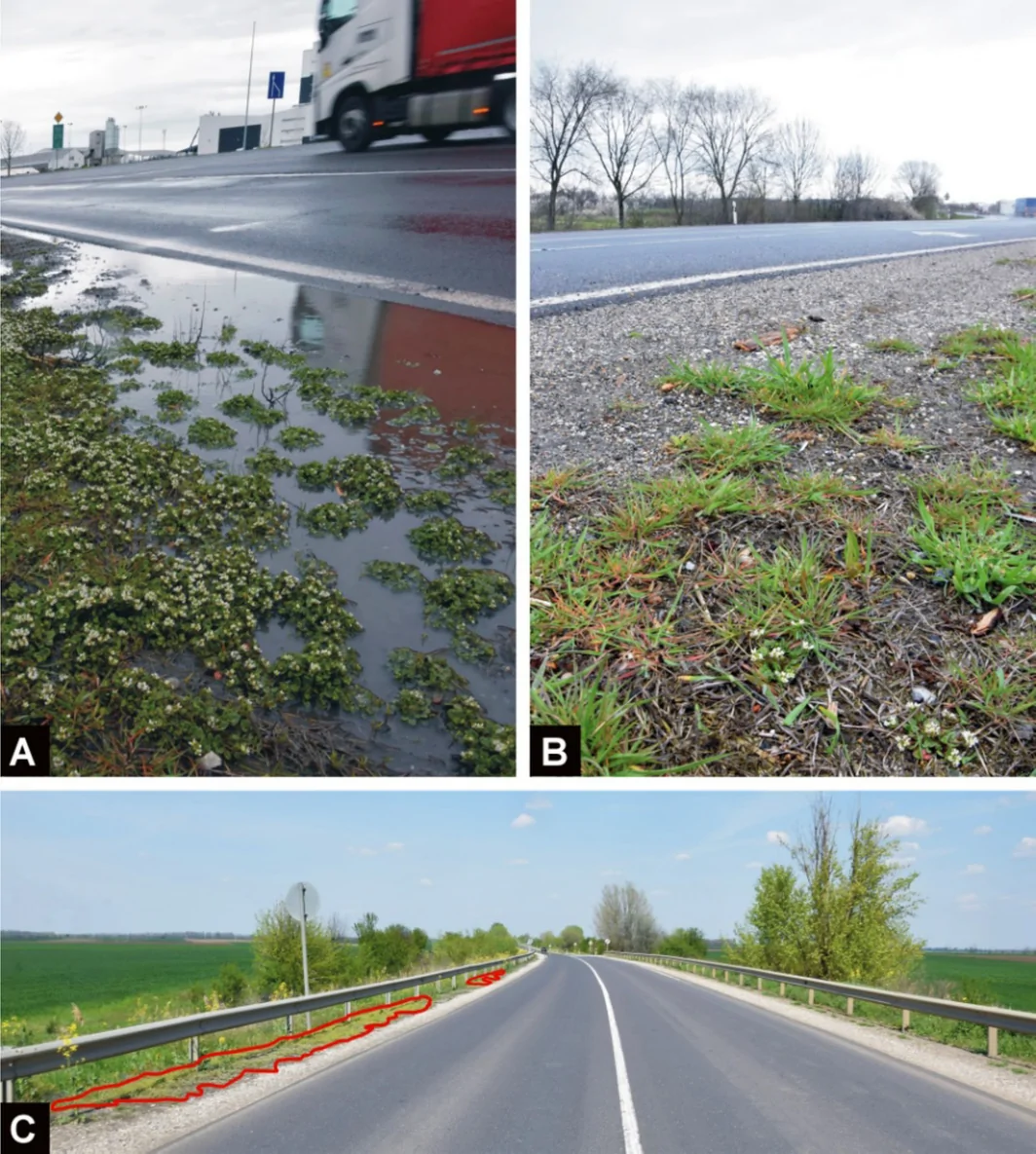
Contrasting roadside conditions on downslope and upslope verges along sloping roads in Hungary. (A, B) Near Biharkeresztes, Hungarian main road No. 42 (E60). (A) The downslope verge receives concentrated road‐surface runoff, associated with higher water availability and a dense cover of *Cochlearia danica* . (B) The opposing upslope verge is drier and sparsely vegetated. Photos A and B were taken at the same location and time on opposite sides of the road, illustrating strong small‐scale asymmetry in moisture supply and *C. danica* abundance. (C) Near Hajdúszoboszló, Hungarian main road No. 4 (E60/E573), showing the transverse camber that channels runoff from both lanes towards the downslope verge. Within the guardrail, the left (downslope) side (outlined in red) forms a ‘green carpet’ of *C. danica* , whereas the upslope side is dominated by pale crushed stone with minimal plant cover.

From an engineering perspective, paved roads are typically constructed with a transverse slope to ensure efficient drainage, directing precipitation laterally towards the road verge (Erlingsson et al. 2008; Awwad 2021). Where road camber or local slope directs runoff predominantly towards one side, opposing verges may experience contrasting moisture conditions (Figure 1). While surface runoff is known to facilitate seed transport on slopes (De Rouw et al. 2018; Bajwa et al. 2018), its indirect effects on roadside soil moisture and plant establishment remain poorly understood.

*Cochlearia danica* L. is an annual halophyte with an Atlantic distribution, historically confined to coastal habitats and saline inland environments of north‐western Europe (Chater and Heywood 1964). Over recent decades, the species has expanded rapidly along salted road networks into temperate continental regions, including Germany, Austria, the Czech Republic, Switzerland, Hungary (Fekete et al. 2018) and Slovakia (Kantor et al. 2026). Notably, several inland populations occur in regions characterised by lower winter–spring precipitation than the species' native Atlantic range, suggesting that local roadside conditions may partly compensate for otherwise marginal macroclimatic conditions (Molnár, Kis, et al. 2024; Süveges 2025).

Here, we investigated whether slope‐driven runoff from paved roads is associated with small‐scale differences in roadside soil‐moisture and abundance of *Cochlearia danica* under continental macroclimatic conditions. Specifically, we tested the hypothesis that downslope verges receiving concentrated runoff exhibit higher soil‐moisture and harbour larger populations than upslope verges. Paired roadside comparisons were used to evaluate whether the observed patterns are consistent with a road‐induced ecohydrological mechanism. Given the species' ongoing expansion, we also surveyed adjacent regions to assess its current distribution and potential establishment beyond its previously known range.

## 2 Materials and Methods

Targeted roadside surveys were conducted between 2023 and 2025 in Hungary, Slovakia, Serbia and Romania (Tables S1 and S2), focusing on motorways and main roads where winter de‐icing is routinely applied. Survey points were established at intervals of approximately 4–5 km where safe stopping was possible. On motorways, surveys were restricted to rest areas and petrol stations in accordance with traffic regulations. Consequently, the survey design was targeted and not fully systematic. Therefore, absence records, particularly along motorways and in regions where stopping opportunities were sparse, should be interpreted cautiously, as small or recently established populations may have remained undetected between survey points. At each site, the presence or absence of *Cochlearia danica* was recorded, along with geographic coordinates, altitude and estimated population size. The geographic distribution of surveyed sampling points is shown in Figure S3. The sampling sites examined in Slovakia followed the roadside survey network reported in Molnár et al. (2026), allowing us to assess whether *C. danica* was present along comparable winter‐salted road sections.

To assess local persistence, selected populations in Hungary and Romania were revisited in 2025 and compared with earlier records from 2023 to 2024 (Molnár, Kis, et al. 2024) to evaluate local persistence.

To examine potential hydrological asymmetry caused by road slope, soil sampling was conducted at sites where road design clearly directed runoff predominantly towards one verge. At 11 sampling sites, composite soil samples (> 1 kg per verge) were collected from both the downslope (runoff‐affected) and upslope (relatively drained) sides. Where *C. danica* was present samples were collected within a radius of approximately 20 cm around plant individuals. Where the species was absent from the upslope verge, samples were collected from the corresponding position opposite the downslope sampling point. Ten subsamples were combined to produce one composite sample from each verge. Composite samples were placed in sealed nylon bags and transported to the laboratory.

Soil‐moisture was quantified gravimetrically at these paired roadside sites where precipitation runoff from the pavement was directed predominantly towards one side of the road. This configuration allowed paired comparison of downslope (runoff‐affected) and upslope (relatively drained) roadside verges within the same site.

All paired soil samples were collected between 15 and 21 April 2023. During this period, precipitation was relatively limited: the nearest meteorological stations recorded 1.1 mm at Túrkeve, 4.5 mm at Szeged and 9.8 mm at Debrecen (HungaroMet Nonprofit Zrt. 2024).

Composite samples were processed within 24 h after collection. In the laboratory, 1000 g measurement samples were weighed from the composite sample, oven‐dried at 105°C for 24 h and then reweighed. This procedure was repeated for samples collected from both sides of the road at all 11 sampling sites. Gravimetric soil‐moisture content (%) was calculated as [(wet sample mass − oven‐dried sample mass)/oven‐dried sample mass] × 100. To facilitate within‐site comparison and to reduce the influence of short‐term weather variability, soil‐moisture was analysed primarily as relative moisture content between paired roadside verges.

Differences in soil‐moisture between downslope and upslope verges were assessed using Wilcoxon's paired test available in R v4.2.1 (R Core Team 2022). The relationship between *Cochlearia danica* population size and relative soil‐moisture or roadside‐position effect was explored separately in two negative binomial regression models for count data implemented in the ‘glmmTMB’ package (Brooks et al. 2017; McGillycuddy et al. 2025) to avoid model overparameterisation due to small sample sizes. Model diagnostics implemented in the ‘DHARMa’ package (Hartig 2024) indicated an adequate fit of the selected models.

To place observed occurrences into a broader climatic context, supplementary species distribution models (SDMs) were generated using occurrence data (*n* = 72,741) from GBIF (GBIF Secretariat 2025), the Pladias Database of the Czech Flora and Vegetation (Chytrý et al. 2021), and Hungarian records (Fekete et al. 2018; Molnár, Kis, et al. 2024; Süveges 2025; Takács et al. 2025). Bioclimatic variables and above sea level elevation from WorldClim were used to characterise macroclimatic suitability (Fick and Hijmans 2017). All environmental layers and spatial thinning were prepared in QGIS v3.44.2 (QGIS 2026). Species distribution models were run in Maxent (Phillips et al. 2025).

For spatial thinning and the filtering of duplicate records, we generated a grid in QGIS with a 2.5‐min resolution corresponding to the WorldClim grid cells and removed multiple occurrences located within the same cell. As a result, each cell retained only one occurrence record. This procedure reduced the influence of spatial clustering and sampling bias in the occurrence dataset (Kramer‐Schadt et al. 2013; Boria et al. 2014).

We conducted a Pearson correlation analysis on the WorldClim variables to further mitigate multicollinearity (Braunisch et al. 2013). Any variables demonstrating a correlation exceeding the |*r*| > 0.7 threshold proposed by Dormann et al. (2013) were not considered (BIO4, BIO6, BIO11, BIO13, BIO14, BIO16, BIO17 and BIO18).

Predictor selection is an important component of SDM development because the included variables should reflect the ecology of the focal species (Merow et al. 2013). In the present study, we prioritised variables relevant to the active growing period of *C. danica* , an autumn‐ or winter‐germinating annual species that undergoes senescence in May. Variables describing climatic conditions outside this active period, such as the maximum temperature of the warmest month (BIO5) and precipitation of the driest month (BIO14), were considered less relevant and were not retained. This parsimonious approach was intended to avoid unnecessary model complexity, which may reduce transferability when models are projected to novel areas (Warren and Seifert 2011). Therefore, we selected temperature annual range (BIO7), annual precipitation (BIO12), precipitation of the coldest quarter (BIO19) and elevation as environmental layers.

To execute the SDM models, we utilised spatially thinned distribution records (*n* = 9672). However, the Pearson correlation analysis indicated a strong correlation between BIO12 and BIO19 (*r* = 0.954), therefore, we analysed them in separate models. The first model incorporated BIO7 and BIO12 variables, whilst the second model retained BIO7 and BIO19. In a third model, BIO7 and elevation were used. During model configuration, we conducted 10 replications per model, applied a regularisation multiplier of 2, established a maximum of 50,000 background points and enabled cross‐validation (Phillips et al. 2025).

For model evaluation, the mean AUC (area under the receiver operating characteristic curve) of the model was used as a measure of predictive accuracy. The relative contributions of each bioclimatic variable to the models were presented as normalised percentages.

In the present study, the SDM was used as a supplementary analysis, primarily identifying whether the newly observed roadside populations occur in areas that are climatically marginal relative to the broader occurrence dataset.

## 3 Results

*Cochlearia danica* was detected at 33 sites across the surveyed region but was not found at the Slovak roadside sites included in our surveys. In addition to its previously documented presence in Hungary, the species was recorded for the first time in Serbia and Romania. Details of newly recorded occurrences in Serbia and Romania are summarised in Table S1, with photographic documentation provided in Figure S1. In Romania, population size increased between 2023 and 2025, suggesting local establishment. Changes in population size between survey years are illustrated in Figure S2.

**FIGURE 2:**
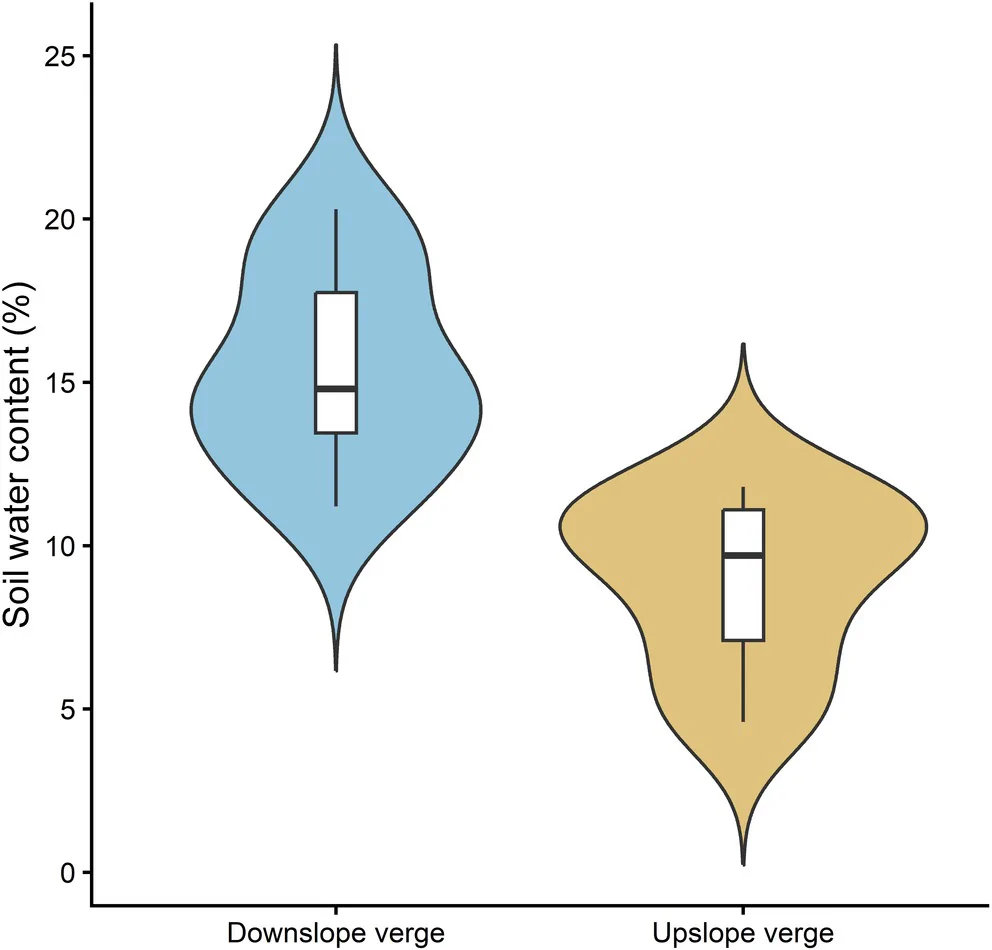
Gravimetric soil‐moisture content on downslope (runoff‐affected) and upslope (drained) roadside verges. Boxes represent median and interquartile range; whiskers indicate the full range of values without outliers. Downslope verges consistently exhibit higher relative soil‐moisture across all paired sites.

**FIGURE 3:**
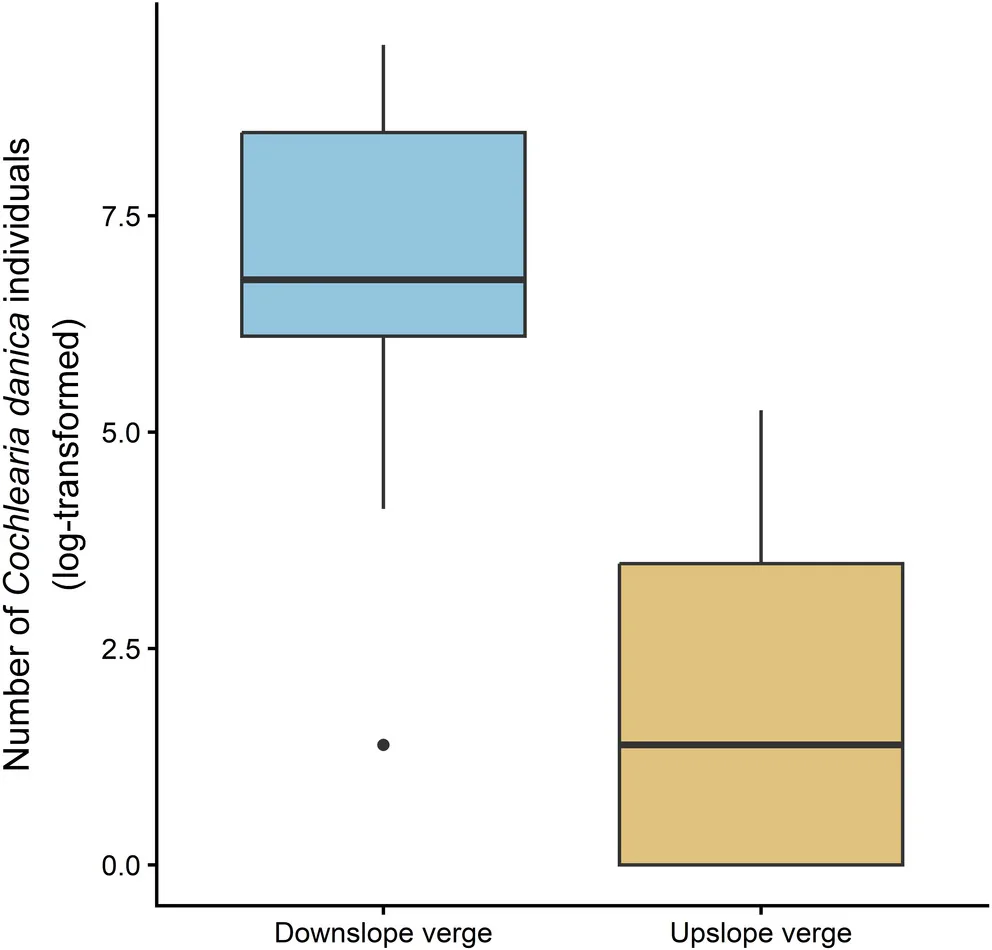
Population size of *Cochlearia danica* on downslope and upslope roadside verges. Population estimates are shown for paired sites. Downslope verges generally harboured substantially larger populations than upslope verges.

**FIGURE 4:**
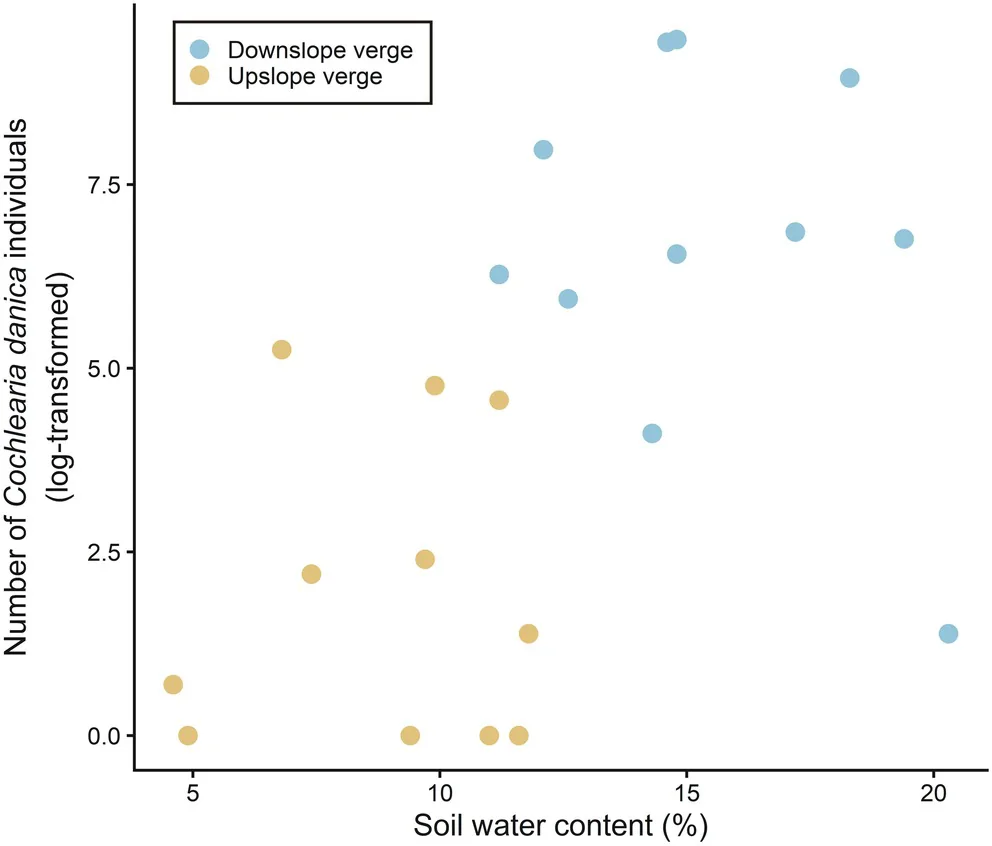
Relationship between gravimetric soil‐moisture content and population size of *Cochlearia danica* on downslope and upslope roadside verges. The figure illustrates the positive association between within‐site soil‐moisture and estimated number of individuals across sampled sites.

At all sites where road slope allowed paired comparison, downslope verges showed consistently higher gravimetric soil‐moisture than upslope verges (Wilcoxon's test: *V* = 66, *p* = 0.004; Figure 2; Table 1). Soil water content ranged from 11.2% to 20.3% on downslope verges and from 4.6% to 11.8% on upslope verges. Higher gravimetric soil‐moisture content was associated with larger *C. danica* populations on runoff‐affected verges (*β* = 0.52, *p* < 0.001). Population sizes of *C. danica* were significantly lower on upslope verges than on downslope verges (*β* = −4.54, *p* < 0.001; Figure 3), corresponding to an approximately 98.9% lower expected abundance on upslope verges. Population size showed a positive association with relative soil‐moisture differences across sites (Figure 4).

**TABLE 1:** Sampling points where soil samples were collected and population estimates were made on both sides of sloping road sections.

ID	Locality	Date	Soil water content (%)		Number of *Cochlearia danica* individuals	
			Downslope	Upslope	Downslope	Upslope
Hu‐014	Hu: Biharkeresztes	2023.04.15	14.8	6.8	862	116
Hu‐015	Hu: Biharkeresztes	2023.04.15	14.3	11	12,540	8
Hu‐016	Hu: Biharkeresztes	2023.04.15	12.6	4.6	13,000	190
Hu‐021	Hu: Ártánd	2023.04.15	19.4	9.9	60	0
Hu‐026	Hu: Berettyóújfalu	2023.04.15	14.6	7.4	380	1
Hu‐041	Hu: Földes	2023.04.15	18.3	11.8	7700	3
Hu‐042	Hu: Földes	2023.04.15	17.2	11.2	945	95
Hu‐056	Hu: Kisújszállás	2023.04.21	14.8	4.9	2900	10
Hu‐100	Hu: Hajdúszoboszló	2023.04.21	12.1	9.7	700	0
Hu‐106	Hu: Püspökladány	2023.04.21	11.2	9.4	530	0
Ro‐79	Ro: Pecica	2023.04.20	20.3	11.6	6	0

*Note:* Downslope refers to the verge receiving concentrated road‐surface runoff; Upslope refers to the opposing, relatively drained verge. Location numbering follows Table S1.

Abbreviations: Hu, Hungary; Ro, Romania.

The first SDM, including temperature annual range (BIO7) and annual precipitation (BIO12), yielded a mean AUC value of 0.873. Among the two environmental variables, BIO7 was the dominant predictor, contributing 93.6% to the model, while BIO12 contributed only 6.4%. The second model performed similarly (AUC = 0.877), in which the percent contribution of BIO19 was 7%. In the third model, including BIO7 and elevation layers, the percent contribution of elevation was substantially high (24.3%) and the model improved (AUC = 0.886) compared to the previous ones. All the SDMs indicated low predicted macroclimatic suitability across the study area in Central Europe for *C. danica* .

**FIGURE 5:**
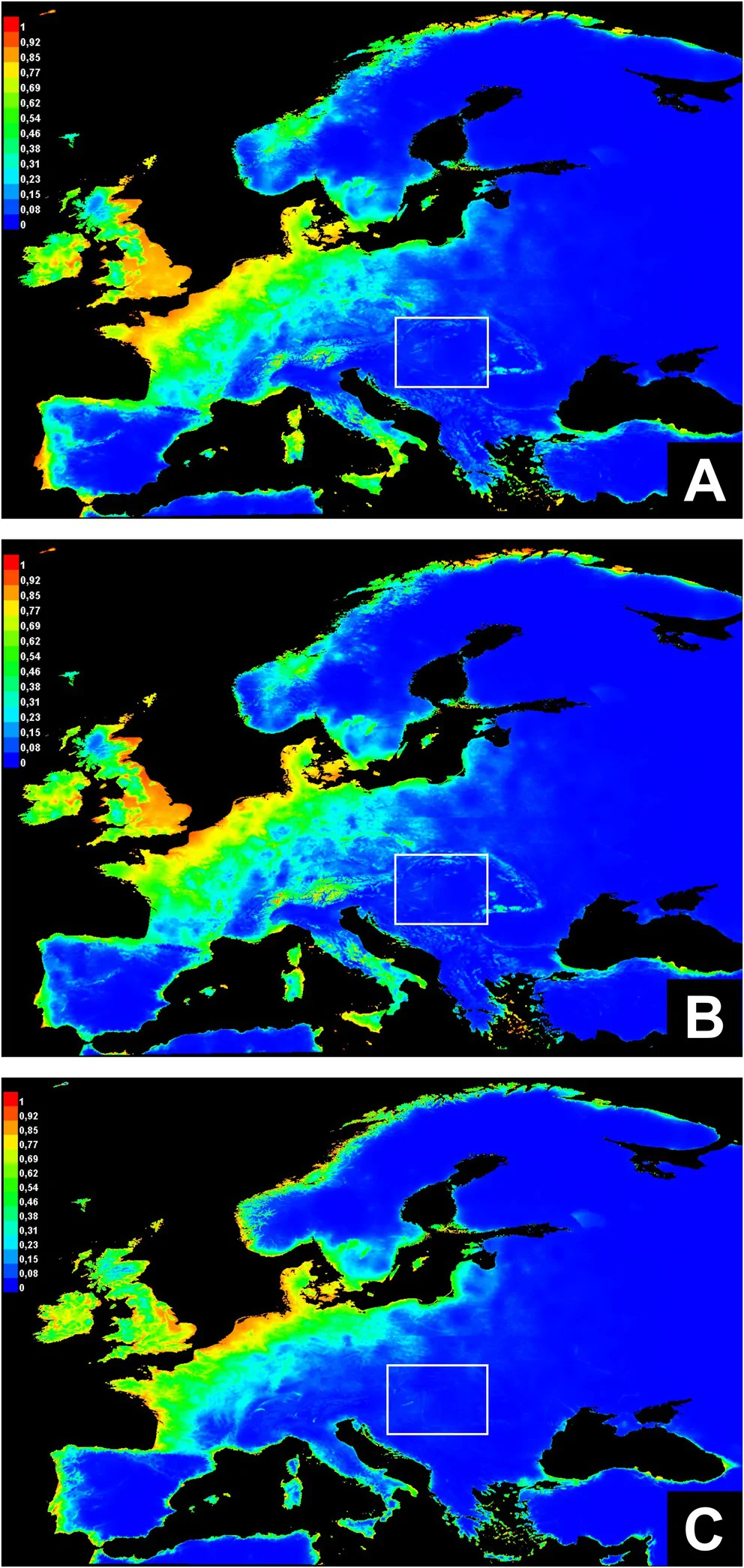
Predicted macroclimatic suitability of *Cochlearia danica* based on species distribution modelling. Panel A shows suitability predicted by the first model including temperature annual range (BIO7) and annual precipitation (BIO12). Panel B shows suitability predicted by the second model including BIO7 and precipitation of the coldest quarter (BIO19). Panel C shows the predicted suitability of the third model used with BIO7 and elevation. The maps represent broad macroclimatic suitability and not fine‐scale roadside habitat suitability predictions. The white rectangle in both panels delineates the boundary of the study area.

These results suggest that the recorded roadside populations of the species occur in regions that are climatically marginal relative to the broader occurrence dataset (Figure 5). However, it should be noted that we performed SDMs as supplementary analyses that did not represent estimates of fine‐scale roadside habitat suitability, as modelling such local conditions was beyond the scope of our study.

## 4 Discussion

The influence of paved road infrastructure on plant dispersal has been extensively documented, particularly with respect to vehicle‐mediated propagule transport and roadside disturbance (Zwaenepoel et al. 2006; von der Lippe et al. 2013; Ansong and Pickering 2013). In contrast, the indirect effects of road design on local abiotic conditions have received considerably less attention. Our results suggest that slope‐driven runoff from paved roads is associated with consistent small‐scale differences in roadside soil‐moisture and may contribute to the establishment and persistence of *Cochlearia danica* in continental roadside habitats. Because our study is observational and soil‐moisture was measured at a limited number of sites, we interpret this mechanism as plausible and biologically consistent rather than definitively demonstrated.

Across all paired sites, downslope verges receiving concentrated runoff consistently exhibited higher relative soil‐moisture than upslope verges. These moisture differences were associated with markedly larger populations of *C. danica* on runoff‐affected sides. Although our study is based on observational data and a limited number of measurements, the pattern across multiple sites and regions is consistent with the interpretation that lateral redistribution of precipitation by road surfaces may create locally favourable conditions for plant establishment. By sampling in the immediate vicinity of *C. danica* individuals, we characterised occupied microsites, but this approach may overrepresent favourable establishment sites. Therefore, the observed moisture–abundance relationship should be interpreted as an association between occupied microsites and roadside hydrological asymmetry.

Road slope and pavement design are fundamental elements of road engineering, intended to ensure rapid drainage of precipitation away from the carriageway (Erlingsson et al. 2008; Awwad 2021). Previous studies have demonstrated that the presence of road pavement influences the distribution of certain plant species (Barbosa et al. 2010; Joly et al. 2011). As a consequence, rain falling on the road surface may be redirected laterally towards one verge where road camber or local slope creates a dominant runoff direction, while infiltration through the pavement itself remains minimal. Similar hydrological asymmetries have been described along unpaved roads, where soil compaction generates linear flow paths and localised water accumulation (Ramos‐Scharrón and LaFevor 2016). Our findings indicate that comparable, though structurally different, processes operate along paved roads, potentially enhancing water availability in adjacent soils on the downslope side.

Based on the records of the closest meteorological stations to the surveyed sites, for the period of 2004–2023, annual precipitation was 568 ± 144 mm (mean ± SD) at Túrkeve, 548 ± 124 mm at Szeged and 544 ± 137 mm at Debrecen (HungaroMet Nonprofit Zrt. 2024). By comparison, mean annual precipitation at six reference localities across the species' native Atlantic coastal distribution ranged from 750 ± 110 mm at Øsløs, Denmark, to 1408 ± 304 mm at Venade, Portugal (Table S3), based on E‐OBS gridded observational data for the same period (Cornes et al. 2018). These values illustrate the substantially drier climatic conditions under which the studied continental roadside populations occur.

For *C. danica* , a halophytic annual species adapted to oceanic climates, such local moisture enhancement may be particularly important. Moisture derived from road runoff may therefore locally alleviate water limitation and contribute to establishment under continental climatic conditions. In addition to increased water availability, runoff may also facilitate the downslope transport of propagules from the road surface, further reinforcing population asymmetry between opposing verges (Bajwa et al. 2018; De Rouw et al. 2018).

Species distribution modelling based on macroclimatic variables predicted low suitability for the study region, particularly within the Pannonian Basin. Nevertheless, several persistent and locally increasing populations of *C danica* were recorded in these areas. This apparent mismatch illustrates the limitations of coarse macroclimatic models in landscapes strongly modified by infrastructure. Roads verges may provide fine‐scale anthropogenic microsites that are not captured by broad climatic predictors, potentially helping to explain the occurrence of *C. danica* in climatically marginal regions. Similar mismatches between native range and observed occurrences have been reported for other road‐associated halophytes and pioneer species (Süveges et al. 2021; Fekete et al. 2022). This pattern should not be interpreted as evidence that roads override macroclimatic constraints, but rather as an indication that coarse climatic models do not represent fine‐scale roadside conditions.

Road verges may function as human‐made niches in which local combinations of runoff, salt input, disturbance and substrate conditions differ substantially from the surrounding landscape matrix.

The detection of *C. danica* in Serbia and Romania represents a further eastward and southward extension of its known continental range. In Romania, the observed increase in population size between survey years suggests successful local establishment. In Serbia, motorway networks connect directly to regions predicted to be climatically more suitable, including areas closer to the Black Sea coast. Since populations continue to expand along these corridors, long‐term monitoring will be needed to determine whether these newly detected populations represent the beginning of broader regional spread, as previously observed in other parts of Central Europe (Fekete et al. 2018; Schmidt et al. 2023).

To sum up, our results suggest that slope‐driven runoff from paved roads is associated with locally increased moisture availability and higher abundance of *C. danica* under continental climatic conditions. More broadly, our findings underscore the importance of considering road‐induced ecohydrological processes when assessing plant range dynamics in increasingly infrastructure‐dominated landscapes.

## 5 Limitations

First, soil‐moisture was assessed at a limited number of paired sites and represents single‐time measurements rather than continuous temporal dynamics. Repeated measurements or long‐term soil‐moisture monitoring would be necessary to confirm whether the observed asymmetry persists across rainfall events and seasons. Because sampling was centred on occupied microsites where the species was present, the measurements may overrepresent locally favourable conditions and do not allow us to distinguish between microsite selection and the potential effect of runoff on establishment. Second, because *Cochlearia danica* is a halophytic species spreading along winter‐salted roads, soil salinity, sodium concentration and electrical conductivity are likely to be important components of roadside habitat suitability. These variables were not quantified in the present study, and therefore the effects of moisture availability and salt accumulation cannot be fully separated. Third, other factors, such as traffic intensity, road maintenance, substrate properties, verge structure and microtopography, may covary with road slope and runoff direction. We therefore interpret slope‐driven runoff as one plausible component of a broader roadside habitat complex, rather than as the sole driver of establishment. Future studies combining high‐resolution hydrological measurements, soil chemistry and experimental approaches would help to estimate the relative importance of these interacting mechanisms.

Roadside occurrences of spreading plant species may vary among years. Although *C. danica* was not detected at the surveyed Slovak roadsides, the species was subsequently reported from Slovak motorways in 2025 (Kantor et al. 2026). Our results should therefore be interpreted as survey‐based non‐detections rather than as evidence of true regional absence.

Despite these limitations, we believe that the observed patterns are spatially consistent and biologically plausible.

## 6 Conclusions

Our study suggests that slope‐driven runoff from paved roads is associated with locally higher roadside soil‐moisture and is associated with increased abundance of *Cochlearia danica* under unfavourable continental climatic conditions.

We propose that roadside environments may function as microrefugia, potentially providing viable habitats for plant species even when broader macroclimatic conditions are unfavourable for their establishment. Road surfaces acting as hydrological modifiers may contribute to the formation of such microrefugia. By documenting new occurrences in Serbia and Romania, we confirm the ongoing continental expansion of this Atlantic halophyte and provide evidence of local establishment beyond its historically oceanic distribution range. Roadside occurrences may depend not only on local hydrological conditions and macroclimate but also on road salting, propagule pressure, traffic connectivity, verge structure, maintenance regime and detection opportunities. The presence or absence of plant species along roadsides can vary dynamically depending on local conditions.

Our findings emphasise that paved roads may facilitate plant range expansion not only by acting as dispersal corridors, but also by creating fine‐scale ecohydrological conditions that are not captured by coarse macroclimatic models. Such infrastructure‐mediated ecohydrological effects may play an important role in shaping species distributions in landscapes dominated by transport networks. Future studies combining repeated soil‐moisture measurements, soil‐chemical analyses and experimental approaches will be needed to quantify the relative importance of runoff, salinity and other roadside factors.

## Author Contributions

**Szabolcs Kis:** investigation (equal), methodology (equal), writing – original draft (equal), writing – review and editing (equal). **Jenő Nagy:** conceptualization (equal), formal analysis (equal), methodology (equal), writing – review and editing (equal). **Attila Molnár V.:** conceptualization (equal), investigation (equal), methodology (equal), supervision (lead), visualization (equal), writing – original draft (equal), writing – review and editing (equal).

## Funding

The research was supported by the National Research, Development and Innovation Office of Hungary (MEC N 24 148930) and by the University of Debrecen Program for Scientific Publication.

## Conflicts of Interest

The authors declare no conflicts of interest.

## Supporting information


**Table S1:** Complete list of recorded occurrences of *Cochlearia danica* in Hungary, Serbia and Romania between 2023 and 2025. The table includes geographic coordinates, survey dates, road characteristics, altitude and estimated population sizes for all detected populations.
**Table S2:** Surveyed roadside sampling points where *Cochlearia danica* was not detected. The table provides location details, survey dates, road types and altitudes for all absence records, ensuring transparency of the sampling design.
**Table S3:** Annual precipitation totals (mm) at three meteorological stations representing the surveyed continental roadside sites in Hungary and at six reference localities across the native Atlantic coastal range of *Cochlearia danica* during 2004–2023. Summary statistics are provided as the mean, median, standard deviation (SD), minimum and maximum of the annual precipitation totals. Hungarian station data were obtained from HungaroMet Nonprofit Zrt. (2024), whereas precipitation values for the Atlantic reference localities were derived from the E‐OBS gridded observational dataset (Cornes et al. 2018).
**Figure S1:** Documented roadside populations of *Cochlearia danica* newly recorded in Romania and Serbia. Photographs show populations at Pecica (Romania) (A), Ruma (Serbia) (B), and Laćarak (Serbia) (C), illustrating local establishment in regions where the species had not previously been reported.
**Figure S2:** Changes in population size of selected *Cochlearia danica* populations between survey years. The figure compares population estimates from 2023 to 2024 with resurvey data from 2025, indicating persistence and local population growth at several sites. Please note that the scale of each Y axis is different and comparisons between populations should be taken accordingly.
**Figure S3:** Geographic distribution of surveyed roadside sampling points and known occurrences of *Cochlearia danica* in the Pannonian Basin and adjacent regions. Surveyed sampling points with *C. danica* (red circles) and without *C. danica* (open circles) are shown, together with previously published occurrence records (black circles) from Fekete et al. (2018), Schmidt et al. (2023), Süveges (2025) and Takács et al. (2025).

## Data Availability

All data used in the preparation of this manuscript are included in the tables and Appendix S1.
