# Supplementary material for: Paved Roads as Ecohydrological Modifiers: Slope‐Driven Runoff Is Associated With Roadside Establishment of the Atlantic Halophyte Cochlearia danica in Continental Europe
Source: Ecol Evol. 2026 Sep 1;16(9):e74212. doi: 10.1002/ece3.74212 (PMC13531357; doi:10.1002/ece3.74212)
Supplement: Supplementary file 1 [file ECE3-16-e74212-s001.docx]

*Supplementary material*

**Table S1.** Complete list of recorded occurrences of *Cochlearia danica* in Hungary, Serbia, and Romania between 2023 and 2025. The table includes geographic coordinates, survey dates, road characteristics, altitude, and estimated population sizes for all detected populations.

| ID | Country | Settlement | Geocoordinates | Date | Type of road | Road number | Altitude asl (m) | No. of individuals |
| --- | --- | --- | --- | --- | --- | --- | --- | --- |
| Hu-002 | Hungary | Berettyóújfalu | 47.18410°N 21.57411°E | 2023.03.27 | main road | 42 (E60) | 93 | 3500 |
| Hu-004 | Hungary | Mezőpeterd | 47.17339°N 21.60380°E | 2023.03.27 | main road | 42 (E60) | 91 | 15 |
| Hu-012 | Hungary | Biharkeresztes | 47.13577°N 21.69390°E | 2023.03.27 | main road | 42 (E60) | 96 | 25 |
| Hu-013 | Hungary | Biharkeresztes | 47.13555°N 21.69490°E | 2023.03.27 | main road | 42 (E60) | 95 | 200 |
| Hu-014 | Hungary | Biharkeresztes | 47.13497°N 21.69872°E | 2023.03.27 | main road | 42 (E60) | 94 | 13200 |
| Hu-015 | Hungary | Biharkeresztes | 47.14126°N 21.71128°E | 2023.03.27 | main road | 42 (E60) | 95 | 60 |
| Hu-016 | Hungary | Biharkeresztes | 47.14222°N 21.71464°E | 2023.03.27 | main road | 42 (E60) | 95 | 380 |
| Hu-018 | Hungary | Biharkeresztes | 47.13927°N 21.73203°E | 2023.03.27 | main road | 42 (E60) | 99 | 2700 |
| Hu-021 | Hungary | Ártánd | 47.12882°N 21.75794°E | 2023.03.27 | main road | 42 (E60) | 101 | 1000 |
| Hu-022 | Hungary | Ártánd | 47.12805°N 21.76222°E | 2023.03.27 | main road | 42 (E60) | 101 | 1700 |
| Hu-026 | Hungary | Berettyóújfalu | 47.18138°N 21.57938°E | 2023.03.27 | main road | 42 (E60) | 91 | 13000 |
| Hu-030 | Hungary | Berettyóújfalu | 47.21261°N 21.56293°E | 2023.03.27 | main road | 42 (E60) | 89 | 3 |
| Hu-041 | Hungary | Földes | 47.28198°N 21.36241°E | 2023.03.29 | main road | 42 (E60) | 84 | 14000 |
| Hu-042 | Hungary | Földes | 47.28627°N 21.33684°E | 2023.03.29 | main road | 42 (E60) | 85 | 1000 |
| Hu-044 | Hungary | Báránd | 47.29810°N 21.20816°E | 2023.03.29 | main road | 42 (E60) | 85 | 1 |
| Hu-056 | Hungary | Kisújszállás | 47.23781°N 20.69358°E | 2023.03.29 | main road | 4 (E60) | 80 | 800 |
| Hu-100 | Hungary | Hajdúszoboszló | 47.43091°N 21.44893°E | 2023.04.21 | main road | 4 (E60) | 93 | 3000 |
| Hu-106 | Hungary | Püspökladány | 47.31758°N 21.02857°E | 2023.04.21 | main road | 4 (E60) | 82 | 530 |
| Hu-158 | Hungary | Ártánd | 47.12702°N 21.76583°E | 2025.04.04 | main road | 42 (E60) | 103 | 8900 |
| Hu-159 | Hungary | Mezőpeterd | 47.17557°N 21.59652°E | 2025.04.04 | main road | 43 (E60) | 92 | 27000 |
| Hu-160 | Hungary | Berettyóújfalu | 47.24338°N 21.50080°E | 2025.04.04 | main road | 44 (E60) | 91 | 500 |
| Hu-165 | Hungary | Földes | 47.27703°N 21.37797°E | 2025.04.04 | main road | 45 (E60) | 88 | 220 |
| Hu-167 | Hungary | Tetétlen | 47.28197°N 21.29552°E | 2025.04.04 | main road | 46 (E60) | 86 | 600 |
| Hu-171 | Hungary | Püspökladány | 47.30246°N 21.18840°E | 2025.04.04 | main road | 47 (E60) | 83 | 76 |
| Hu-172 | Hungary | Püspökladány | 47.30230°N 21.17558°E | 2025.04.04 | main road | 48 (E60) | 84 | 1800 |
| Hu-174 | Hungary | Püspökladány | 47.30936°N 21.13929°E | 2025.04.04 | main road | 49 (E60) | 85 | 7 |
| Hu-180 | Hungary | Karcag | 47.29837°N 20.93212°E | 2025.04.04 | main road | 4 (E60) | 84 | 400 |
| Hu-182 | Hungary | Kiskunhalas | 46.37981°N 19.50919°E | 2025.04.08 | main road | 53 | 125 | 100 |
| Hu-183 | Hungary | Balotaszállás | 46.36330°N 19.51882°E | 2025.04.08 | main road | 53 | 130 | 30 |
| Ro-79 | Romania | Pecica | 46.17896°N 21.11551°E | 2023.04.20 | motorway | A1 (E68) | 100 | 6 |
| Srb-26 | Serbia | Ruma | 44.94419°N 19.86624°E | 2023.04.12 | motorway | A3 (E70) | 81 | 200 |
| Srb-37 | Serbia | Laćarak | 45.01871°N 19.52234°E | 2023.04.13 | motorway | A3 (E70) | 77 | 73 |
| Srb-45 | Serbia | Beograd | 44.91988°N 20.30455°E | 2023.04.13 | motorway | A1 (E75) | 96 | 20 |

**Table S2**. Surveyed roadside sampling points where Cochlearia danica was not detected. The table provides location details, survey dates, road types, and altitudes for all absence records, ensuring transparency of the sampling design.

| ID | Country | Settlement | Geocoordinates | Date | Type of road | Altitude asl (m) |
| --- | --- | --- | --- | --- | --- | --- |
| Hu-001 | Hungary | Berettyóújfalu | 47.22654°N 21.55425°E | 2023.03.27 | main road | 92 |
| Hu-003 | Hungary | Berettyóújfalu | 47.18526°N 21.57343°E | 2023.03.27 | main road | 92 |
| Hu-005 | Hungary | Mezőpeterd | 47.16926°N 21.61540°E | 2023.03.27 | main road | 92 |
| Hu-006 | Hungary | Mezőpeterd | 47.16544°N 21.62814°E | 2023.03.27 | main road | 91 |
| Hu-007 | Hungary | Biharkeresztes | 47.16045°N 21.66392°E | 2023.03.27 | secondary road | 93 |
| Hu-008 | Hungary | Biharkeresztes | 47.14485°N 21.67384°E | 2023.03.27 | main road | 92 |
| Hu-009 | Hungary | Biharkeresztes | 47.15062°N 21.66151°E | 2023.03.27 | main road | 91 |
| Hu-010 | Hungary | Biharkeresztes | 47.14485°N 21.67384°E | 2023.03.27 | main road | 92 |
| Hu-011 | Hungary | Biharkeresztes | 47.14063°N 21.68326°E | 2023.03.27 | main road | 94 |
| Hu-017 | Hungary | Biharkeresztes | 47.13970°N 21.72966°E | 2023.03.27 | main road | 99 |
| Hu-019 | Hungary | Ártánd | 47.13009°N 21.74930°E | 2023.03.27 | main road | 101 |
| Hu-020 | Hungary | Biharkeresztes | 47.13021°N 21.74784°E | 2023.03.27 | main road | 101 |
| Hu-023 | Hungary | Ártánd | 47.12439°N 21.77365°E | 2023.03.27 | main road | 102 |
| Hu-024 | Hungary | Ártánd | 47.12028°N 21.78588°E | 2023.03.27 | main road | 103 |
| Hu-025 | Hungary | Berettyóújfalu | 47.17704°N 21.59217°E | 2023.03.27 | main road | 90 |
| Hu-027 | Hungary | Berettyóújfalu | 47.18742°N 21.56044°E | 2023.03.27 | main road | 92 |
| Hu-028 | Hungary | Berettyóújfalu | 47.18745°N 21.57254°E | 2023.03.27 | main road | 92 |
| Hu-029 | Hungary | Berettyóújfalu | 47.19734°N 21.57254°E | 2023.03.27 | main road | 93 |
| Hu-031 | Hungary | Berettyóújfalu | 47.22197°N 21.55788°E | 2023.03.27 | main road | 90 |
| Hu-032 | Hungary | Debrecen | 47.47485°N 21.63453°E | 2023.03.29 | main road | 104 |
| Hu-033 | Hungary | Mikepércs | 47.43316°N 21.62754°E | 2023.03.29 | main road | 101 |
| Hu-034 | Hungary | Sáránd | 47.39510°N 21.61356°E | 2023.03.29 | main road | 98 |
| Hu-035 | Hungary | Derecske | 47.33612°N 21.56220°E | 2023.03.29 | main road | 94 |
| Hu-036 | Hungary | Tépe | 47.30247°N 21.55682°E | 2023.03.29 | main road | 91 |
| Hu-037 | Hungary | Berettyóújfalu | 47.26805°N 21.54702°E | 2023.03.29 | main road | 90 |
| Hu-038 | Hungary | Berettyóújfalu | 47.24033°N 21.51136°E | 2023.03.29 | main road | 92 |
| Hu-039 | Hungary | Berettyóújfalu | 47.25574°N 21.45519°E | 2023.03.29 | main road | 90 |
| Hu-040 | Hungary | Földes | 47.27281°N 21.39289°E | 2023.03.29 | main road | 87 |
| Hu-043 | Hungary | Báránd | 47.28545°N 21.27379°E | 2023.03.29 | main road | 85 |
| Hu-045 | Hungary | Püspökladány | 47.30499°N 21.14747°E | 2023.03.29 | main road | 84 |
| Hu-046 | Hungary | Püspökladány | 47.32264°N 21.07899°E | 2023.03.29 | main road | 84 |
| Hu-047 | Hungary | Karcag | 47.31707°N 21.02370°E | 2023.03.29 | main road | 83 |
| Hu-048 | Hungary | Karcag | 47.31002°N 20.95527°E | 2023.03.29 | main road | 84 |
| Hu-049 | Hungary | Karcag | 47.28344°N 20.90259°E | 2023.03.29 | main road | 82 |
| Hu-050 | Hungary | Karcag | 47.26095°N 20.84963°E | 2023.03.29 | main road | 81 |
| Hu-051 | Hungary | Kisújszállás | 47.22975°N 20.80812°E | 2023.03.29 | main road | 82 |
| Hu-052 | Hungary | Kisújszállás | 47.23675°N 20.78190°E | 2023.03.29 | main road | 81 |
| Hu-053 | Hungary | Kisújszállás | 47.23707°N 20.78120°E | 2023.03.29 | main road | 80 |
| Hu-054 | Hungary | Kisújszállás | 47.24471°N 20.75204°E | 2023.03.29 | main road | 81 |
| Hu-055 | Hungary | Kisújszállás | 47.23842°N 20.69874°E | 2023.03.29 | main road | 80 |
| Hu-057 | Hungary | Kisújszállás | 47.23794°N 20.69201°E | 2023.03.29 | main road | 81 |
| Hu-058 | Hungary | Kenderes | 47.24229°N 20.63732°E | 2023.03.29 | main road | 82 |
| Hu-059 | Hungary | Fegyvernek | 47.23657°N 20.57822°E | 2023.03.29 | main road | 82 |
| Hu-060 | Hungary | Törökszentmiklós | 47.22094°N 20.50139°E | 2023.03.29 | main road | 82 |
| Hu-061 | Hungary | Törökszentmiklós | 47.19111°N 20.45143°E | 2023.03.29 | main road | 82 |
| Hu-062 | Hungary | Létavértes | 47.38163°N 21.95432°E | 2023.04.02 | secondary road | 122 |
| Hu-063 | Hungary | Furta | 47.14486°N 21.48810°E | 2023.04.12 | main road | 90 |
| Hu-064 | Hungary | Csökmő | 47.01445°N 21.25417°E | 2023.04.12 | main road | 85 |
| Hu-065 | Hungary | Tótkomlós | 46.44305°N 20.78603°E | 2023.04.12 | secondary road | 89 |
| Hu-066 | Hungary | Makó | 46.35023°N 20.62169°E | 2023.04.12 | secondary road | 85 |
| Hu-067 | Hungary | Makó | 46.33607°N 20.59343°E | 2023.04.12 | secondary road | 84 |
| Hu-068 | Hungary | Makó | 46.23861°N 20.49678°E | 2023.04.12 | main road | 76 |
| Hu-069 | Hungary | Makó | 46.25031°N 20.48524°E | 2023.04.12 | main road | 79 |
| Hu-070 | Hungary | Óföldeák | 46.27467°N 20.43839°E | 2023.04.12 | motorway | 75 |
| Hu-071 | Hungary | Algyő | 46.28555°N 20.28666°E | 2023.04.12 | motorway | 74 |
| Hu-072 | Hungary | Algyő | 46.28606°N 20.28513°E | 2023.04.12 | motorway | 74 |
| Hu-073 | Hungary | Szeged | 46.29440°N 20.17991°E | 2023.04.12 | motorway | 81 |
| Hu-074 | Hungary | Szeged | 46.32821°N 20.13299°E | 2023.04.12 | secondary road | 79 |
| Hu-075 | Hungary | Szeged | 46.32688°N 20.13793°E | 2023.04.12 | secondary road | 78 |
| Hu-076 | Hungary | Domaszék | 46.24843°N 20.03601°E | 2023.04.12 | motorway | 82 |
| Hu-077 | Hungary | Domaszék | 46.24896°N 20.03691°E | 2023.04.12 | motorway | 81 |
| Hu-078 | Hungary | Kiskundorozsma | 46.29984°N 20.04304°E | 2023.04.13 | motorway | 81 |
| Hu-079 | Hungary | Hajdúböszörmény | 47.69406°N 21.43882°E | 2023.04.17 | main road | 98 |
| Hu-080 | Hungary | Muhi | 47.96992°N 20.93033°E | 2023.04.17 | main road | 98 |
| Hu-081 | Hungary | Arnót | 48.13546°N 20.88078°E | 2023.04.17 | main road | 126 |
| Hu-082 | Hungary | Forró | 48.30170°N 21.05745°E | 2023.04.17 | main road | 136 |
| Hu-083 | Hungary | Dubicsány | 48.28043°N 20.50541°E | 2023.04.19 | main road | 138 |
| Hu-084 | Hungary | Nyékládháza | 47.98359°N 20.86315°E | 2023.04.19 | main road | 100 |
| Hu-085 | Hungary | Hejőbába | 47.92165°N 20.97566°E | 2023.04.19 | secondary road | 95 |
| Hu-086 | Hungary | Tiszaújváros | 47.92165°N 21.05653°E | 2023.04.19 | main road | 92 |
| Hu-087 | Hungary | Debrecen | 47.49538°N 21.63197°E | 2023.04.20 | main road | 109 |
| Hu-088 | Hungary | Békés | 46.75659°N 21.12475°E | 2023.04.20 | main road | 81 |
| Hu-089 | Hungary | Csanádpalota | 46.21709°N 20.71571°E | 2023.04.20 | motorway | 87 |
| Hu-090 | Hungary | Csanádpalota | 46.21603°N 20.71798°E | 2023.04.20 | motorway | 86 |
| Hu-091 | Hungary | Csanádpalota | 46.21923°N 20.71297°E | 2023.04.20 | secondary road | 87 |
| Hu-092 | Hungary | Kötegyán | 46.76362°N 21.47882°E | 2023.04.20 | secondary road | 87 |
| Hu-093 | Hungary | Kötegyán | 46.76092°N 21.46530°E | 2023.04.20 | secondary road | 88 |
| Hu-094 | Hungary | Méhkerék | 46.75837°N 21.45258°E | 2023.04.20 | secondary road | 87 |
| Hu-095 | Hungary | Sarkad | 46.75567°N 21.44006°E | 2023.04.20 | secondary road | 85 |
| Hu-096 | Hungary | Debrecen | 47.51346°N 21.60404°E | 2023.04.21 | main road | 110 |
| Hu-097 | Hungary | Debrecen | 47.49642°N 21.54325°E | 2023.04.21 | main road | 106 |
| Hu-098 | Hungary | Ebes | 47.47452°N 21.47955°E | 2023.04.21 | main road | 99 |
| Hu-099 | Hungary | Hajdúszoboszló | 47.43917°N 21.44789°E | 2023.04.21 | main road | 96 |
| Hu-101 | Hungary | Hajdúszoboszló | 47.41332°N 21.41884°E | 2023.04.21 | main road | 88 |
| Hu-102 | Hungary | Hajdúszoboszló | 47.42339°N 21.36798°E | 2023.04.21 | main road | 87 |
| Hu-103 | Hungary | Hajdúszoboszló | 47.40184°N 21.31711°E | 2023.04.21 | main road | 89 |
| Hu-104 | Hungary | Kaba | 47.36884°N 21.25780°E | 2023.04.21 | main road | 87 |
| Hu-105 | Hungary | Püspökladány | 47.34556°N 21.18770°E | 2023.04.21 | main road | 86 |
| Hu-107 | Hungary | Karcag | 47.29394°N 20.93322°E | 2023.04.21 | secondary road | 83 |
| Hu-108 | Hungary | Karcag | 47.25163°N 20.94852°E | 2023.04.21 | secondary road | 81 |
| Hu-109 | Hungary | Újfehértó | 47.75528°N 21.67682°E | 2023.05.10 | main road | 124 |
| Hu-110 | Hungary | Újfehértó | 47.76304°N 21.67645°E | 2023.05.10 | main road | 123 |
| Hu-111 | Hungary | Újfehértó | 47.80489°N 21.67802°E | 2023.05.10 | secondary road | 119 |
| Hu-112 | Hungary | Újfehértó | 47.81321°N 21.68751°E | 2023.05.10 | secondary road | 119 |
| Hu-113 | Hungary | Érpatak | 47.80783°N 21.75325°E | 2023.05.10 | secondary road | 127 |
| Hu-114 | Hungary | Érpatak | 47.80866°N 21.74937°E | 2023.05.10 | secondary road | 125 |
| Hu-115 | Hungary | Pocsaj | 47.28214°N 21.85049°E | 2023.05.10 | secondary road | 101 |
| Hu-116 | Hungary | Pocsaj | 47.27958°N 21.85523°E | 2023.05.10 | secondary road | 98 |
| Hu-117 | Hungary | Pocsaj | 47.27909°N 21.85860°E | 2023.05.10 | secondary road | 97 |
| Hu-118 | Hungary | Pocsaj | 47.26718°N 21.87457°E | 2023.05.10 | secondary road | 98 |
| Hu-119 | Hungary | Pocsaj | 47.26807°N 21.87394°E | 2023.05.10 | secondary road | 100 |
| Hu-120 | Hungary | Pocsaj | 47.27365°N 21.87943°E | 2023.05.10 | secondary road | 100 |
| Hu-121 | Hungary | Bocskaikert | 47.60426°N 21.62646°E | 2023.05.23 | secondary road | 132 |
| Hu-122 | Hungary | Bocskaikert | 47.64910°N 21.65028°E | 2023.05.23 | secondary road | 148 |
| Hu-123 | Hungary | Hajdúhadház | 47.68818°N 21.65525°E | 2023.05.23 | secondary road | 144 |
| Hu-124 | Hungary | Nyíregyháza | 47.88721°N 21.74703°E | 2023.05.23 | motorway | 115 |
| Hu-125 | Hungary | Nagykálló | 47.89240°N 21.80042°E | 2023.05.23 | motorway | 120 |
| Hu-126 | Hungary | Nyíregyháza | 47.89582°N 21.81235°E | 2023.05.23 | motorway | 114 |
| Hu-127 | Hungary | Nyíregyháza | 47.90811°N 21.81825°E | 2023.05.23 | secondary road | 118 |
| Hu-128 | Hungary | Nyíregyháza | 47.93528°N 21.81876°E | 2023.05.23 | secondary road | 114 |
| Hu-129 | Hungary | Nyíregyháza | 47.97938°N 21.81877°E | 2023.05.23 | secondary road | 101 |
| Hu-130 | Hungary | Nyírtura | 48.01262°N 21.84404°E | 2023.05.23 | main road | 99 |
| Hu-131 | Hungary | Nyírbogdány | 48.04360°N 21.87207°E | 2023.05.23 | main road | 99 |
| Hu-132 | Hungary | Székely | 48.06630°N 21.93381°E | 2023.05.23 | secondary road | 96 |
| Hu-133 | Hungary | Nyírtass | 48.10528°N 21.98842°E | 2023.05.23 | secondary road | 98 |
| Hu-134 | Hungary | Nyírtass | 48.12645°N 22.01030°E | 2023.05.23 | main road | 98 |
| Hu-135 | Hungary | Ajak | 48.16635°N 22.03306°E | 2023.05.23 | main road | 91 |
| Hu-136 | Hungary | Kisvárda | 48.20618°N 22.05148°E | 2023.05.23 | main road | 93 |
| Hu-137 | Hungary | Kisvárda | 48.24431°N 22.03146°E | 2023.05.23 | main road | 90 |
| Hu-138 | Hungary | Kékcse | 48.24878°N 21.98296°E | 2023.05.23 | main road | 99 |
| Hu-139 | Hungary | Cigánd | 48.26926°N 21.94293°E | 2023.05.23 | main road | 104 |
| Hu-140 | Hungary | Cigánd | 48.27490°N 21.93773°E | 2023.05.23 | secondary road | 95 |
| Hu-141 | Hungary | Sátoraljaújhely | 48.38104°N 21.65209°E | 2023.05.23 | main road | 99 |
| Hu-142 | Hungary | Sátoraljaújhely | 48.37244°N 21.60986°E | 2023.05.23 | secondary road | 92 |
| Hu-143 | Hungary | Bodrogolaszi | 48.28722°N 21.50504°E | 2023.05.23 | secondary road | 114 |
| Hu-144 | Hungary | Vámosújfalu | 48.26015°N 21.43710°E | 2023.05.23 | main road | 112 |
| Hu-145 | Hungary | Olaszliszka | 48.23151°N 21.40336°E | 2023.05.23 | main road | 105 |
| Hu-146 | Hungary | Bodrogkisfalud | 48.18555°N 21.35712°E | 2023.05.23 | main road | 121 |
| Hu-147 | Hungary | Debrecen | 47.55979°N 21.48614°E | 2023.05.23 | main road | 122 |
| Hu-148 | Hungary | Nagyhegyes | 47.56179°N 21.42009°E | 2023.05.23 | main road | 111 |
| Hu-149 | Hungary | Nagyhegyes | 47.55532°N 21.35294°E | 2023.05.23 | main road | 90 |
| Hu-150 | Hungary | Nagyhegyes | 47.56665°N 21.28642°E | 2023.05.23 | main road | 87 |
| Hu-151 | Hungary | Hortobágy | 47.57762°N 21.22730°E | 2023.05.23 | main road | 85 |
| Hu-152 | Hungary | Hortobágy | 47.58179°N 21.13987°E | 2023.05.23 | main road | 85 |
| Hu-153 | Hungary | Hortobágy | 47.59133°N 21.06972°E | 2023.05.23 | main road | 91 |
| Hu-154 | Hungary | Hortobágy | 47.58288°N 21.00472°E | 2023.05.23 | main road | 91 |
| Hu-155 | Hungary | Tiszafüred | 47.55036°N 20.95230°E | 2023.05.23 | main road | 88 |
| Hu-156 | Hungary | Tiszafüred | 47.54790°N 20.89040°E | 2023.05.23 | main road | 90 |
| Hu-157 | Hungary | Tiszafüred | 47.59378°N 20.84539°E | 2023.05.23 | main road | 88 |
| Hu-161 | Hungary | Berettyóújfalu | 47.25013°N 21.47624°E | 2025.04.06 | main road | 90 |
| Hu-162 | Hungary | Berettyóújfalu | 47.25705°N 21.45099°E | 2025.04.06 | main road | 92 |
| Hu-163 | Hungary | Berettyóújfalu | 47.26504°N 21.42180°E | 2025.04.06 | main road | 87 |
| Hu-164 | Hungary | Berettyóújfalu | 47.27011°N 21.40327°E | 2025.04.06 | main road | 87 |
| Hu-166 | Hungary | Földes | 47.28723°N 21.34733°E | 2025.04.06 | main road | 87 |
| Hu-168 | Hungary | Báránd | 47.28526°N 21.27487°E | 2025.04.06 | main road | 86 |
| Hu-169 | Hungary | Báránd | 47.28964°N 21.25351°E | 2025.04.06 | main road | 85 |
| Hu-170 | Hungary | Báránd | 47.29635°N 21.21542°E | 2025.04.06 | main road | 86 |
| Hu-173 | Hungary | Püspökladány | 47.30235°N 21.15277°E | 2025.04.06 | main road | 84 |
| Hu-175 | Hungary | Püspökladány | 47.32192°N 21.07086°E | 2025.04.06 | main road | 83 |
| Hu-176 | Hungary | Püspökladány | 47.31970°N 21.04947°E | 2025.04.06 | main road | 84 |
| Hu-177 | Hungary | Karcag | 47.31407°N 20.99404°E | 2025.04.06 | main road | 84 |
| Hu-178 | Hungary | Karcag | 47.31034°N 20.95864°E | 2025.04.06 | main road | 83 |
| Hu-179 | Hungary | Kiskunhalas | 46.41327°N 19.49575°E | 2025.04.08 | main road | 123 |
| Hu-181 | Hungary | Kiskunhalas | 46.39709°N 19.50151°E | 2025.04.08 | main road | 124 |
| Hu-184 | Hungary | Balotaszállás | 46.34157°N 19.51937°E | 2025.04.08 | main road | 130 |
| Hu-185 | Hungary | Balotaszállás | 46.32044°N 19.52395°E | 2025.04.08 | main road | 134 |
| Hu-186 | Hungary | Kisszállás | 46.30259°N 19.52422°E | 2025.04.08 | main road | 126 |
| Hu-187 | Hungary | Kisszállás | 46.28217°N 19.52895°E | 2025.04.08 | main road | 127 |
| Hu-188 | Hungary | Kisszállás | 46.27074°N 19.53201°E | 2025.04.08 | main road | 125 |
| Hu-189 | Hungary | Kisszállás | 46.24938°N 19.53350°E | 2025.04.08 | main road | 124 |
| Hu-190 | Hungary | Tompa | 46.23690°N 19.53515°E | 2025.04.08 | main road | 127 |
| Hu-191 | Hungary | Tompa | 46.19442°N 19.53713°E | 2025.04.08 | main road | 135 |
| Hu-192 | Hungary | Öttömös | 46.25219°N 19.64957°E | 2025.04.08 | main road | 123 |
| Hu-193 | Hungary | Öttömös | 46.25606°N 19.65995°E | 2025.04.08 | main road | 123 |
| Hu-194 | Hungary | Öttömös | 46.24291°N 19.69953°E | 2025.04.08 | main road | 122 |
| Hu-195 | Hungary | Öttömös | 46.23475°N 19.72913°E | 2025.04.08 | main road | 119 |
| Hu-196 | Hungary | Ásotthalom | 46.22361°N 19.76603°E | 2025.04.08 | main road | 114 |
| Hu-197 | Hungary | Mórahalom | 46.22566°N 19.83472°E | 2025.04.08 | secondary road | 103 |
| Hu-198 | Hungary | Mórahalom | 46.21825°N 19.85473°E | 2025.04.08 | secondary road | 101 |
| Hu-199 | Hungary | Mórahalom | 46.23494°N 19.85605°E | 2025.04.08 | main road | 98 |
| Hu-200 | Hungary | Mórahalom | 46.23669°N 19.87298°E | 2025.04.08 | main road | 94 |
| Hu-201 | Hungary | Mórahalom | 46.23462°N 19.88725°E | 2025.04.08 | main road | 90 |
| Hu-202 | Hungary | Mórahalom | 46.22373°N 19.90908°E | 2025.04.08 | main road | 88 |
| Hu-203 | Hungary | Domaszék | 46.24127°N 20.00371°E | 2025.04.08 | main road | 83 |
| Ro-01 | Romania | Borș | 47.11541°N 21.80447°E | 2023.03.31 | main road | 104 |
| Ro-02 | Romania | Borș | 47.10934°N 21.81884°E | 2023.03.31 | main road | 105 |
| Ro-03 | Romania | Sântion | 47.10431°N 21.83224°E | 2023.03.31 | main road | 107 |
| Ro-04 | Romania | Oradea | 47.09555°N 21.86452°E | 2023.03.31 | main road | 112 |
| Ro-05 | Romania | Oradea | 47.03494°N 21.89908°E | 2023.03.31 | main road | 138 |
| Ro-06 | Romania | Oradea | 47.02255°N 21.89478°E | 2023.03.31 | main road | 136 |
| Ro-07 | Romania | Oradea | 47.01352°N 21.88626°E | 2023.03.31 | main road | 144 |
| Ro-08 | Romania | Nojorid | 46.98943°N 21.87469°E | 2023.03.31 | main road | 146 |
| Ro-09 | Romania | Leş | 46.97260°N 21.84688°E | 2023.03.31 | main road | 126 |
| Ro-10 | Romania | Gepiu | 46.93975°N 21.80270°E | 2023.03.31 | main road | 116 |
| Ro-11 | Romania | Gepiu | 46.93479°N 21.79613°E | 2023.03.31 | main road | 112 |
| Ro-12 | Romania | Gepiu | 46.92251°N 21.78134°E | 2023.03.31 | main road | 108 |
| Ro-13 | Romania | Inand | 46.88367°N 21.74639°E | 2023.03.31 | main road | 103 |
| Ro-14 | Romania | Mădăras | 46.83876°N 21.70601°E | 2023.03.31 | main road | 92 |
| Ro-15 | Romania | Mădăras | 46.82072°N 21.67544°E | 2023.03.31 | main road | 93 |
| Ro-16 | Romania | Salonta | 46.79816°N 21.63762°E | 2023.03.31 | main road | 92 |
| Ro-17 | Romania | Salonta | 46.79529°N 21.62766°E | 2023.03.31 | main road | 92 |
| Ro-18 | Romania | Salonta | 46.79124°N 21.61114°E | 2023.03.31 | main road | 92 |
| Ro-19 | Romania | Salonta | 46.78826°N 21.59708°E | 2023.03.31 | main road | 90 |
| Ro-20 | Romania | Salonta | 46.78573°N 21.58479°E | 2023.03.31 | main road | 89 |
| Ro-21 | Romania | Salonta | 46.78596°N 21.58446°E | 2023.03.31 | main road | 90 |
| Ro-22 | Romania | Salonta | 46.78042°N 21.55914°E | 2023.03.31 | main road | 88 |
| Ro-23 | Romania | Salonta | 46.77786°N 21.54659°E | 2023.03.31 | main road | 87 |
| Ro-24 | Romania | Comuna Ciumeghiu | 46.77511°N 21.53362°E | 2023.03.31 | main road | 86 |
| Ro-25 | Romania | Comuna Ciumeghiu | 46.77264°N 21.52110°E | 2023.03.31 | main road | 85 |
| Ro-26 | Romania | Comuna Ciumeghiu | 46.76985°N 21.50807°E | 2023.03.31 | main road | 85 |
| Ro-27 | Romania | Comuna Ciumeghiu | 46.76732°N 21.49635°E | 2023.03.31 | main road | 86 |
| Ro-28 | Romania | Brătești | 46.76707°N 22.18518°E | 2023.03.31 | main road | 134 |
| Ro-29 | Romania | Sâmbăta | 46.80832°N 22.19123°E | 2023.03.31 | main road | 148 |
| Ro-30 | Romania | Ceica | 46.86824°N 22.16297°E | 2023.03.31 | main road | 166 |
| Ro-31 | Romania | Bucium | 46.88415°N 22.14692°E | 2023.03.31 | main road | 177 |
| Ro-32 | Romania | Dicănești | 46.90695°N 22.11659°E | 2023.03.31 | main road | 281 |
| Ro-33 | Romania | Hidișelu de Sus | 46.93123°N 22.06909°E | 2023.03.31 | main road | 253 |
| Ro-34 | Romania | Hidișelu de Jos | 46.94398°N 22.02940°E | 2023.03.31 | main road | 177 |
| Ro-35 | Romania | Cordău | 46.97041°N 21.98982°E | 2023.03.31 | main road | 163 |
| Ro-36 | Romania | Săcădat | 47.05995°N 22.13703°E | 2023.03.31 | main road | 168 |
| Ro-37 | Romania | Urvind | 47.06623°N 22.29673°E | 2023.03.31 | main road | 252 |
| Ro-38 | Romania | Florești | 46.75209°N 23.52355°E | 2023.04.02 | main road | 358 |
| Ro-39 | Romania | Florești | 46.74432°N 23.46009°E | 2023.04.02 | main road | 379 |
| Ro-40 | Romania | Gilău | 46.76591°N 23.35673°E | 2023.04.02 | main road | 421 |
| Ro-41 | Romania | Căpușu Mare | 46.77369°N 23.32572°E | 2023.04.02 | main road | 447 |
| Ro-42 | Romania | Căpușu Mare | 46.79741°N 23.27677°E | 2023.04.02 | main road | 473 |
| Ro-43 | Romania | Dumbrava | 46.81319°N 23.22921°E | 2023.04.02 | main road | 567 |
| Ro-44 | Romania | Păniceni | 46.82119°N 23.16846°E | 2023.04.02 | main road | 657 |
| Ro-45 | Romania | Izvoru Crișului | 46.83682°N 23.11911°E | 2023.04.02 | main road | 596 |
| Ro-46 | Romania | Șaula | 46.85182°N 23.05787°E | 2023.04.02 | main road | 556 |
| Ro-47 | Romania | Huedin | 46.86951°N 22.99345°E | 2023.04.02 | main road | 528 |
| Ro-48 | Romania | Morlaca | 46.87882°N 22.94111°E | 2023.04.02 | main road | 519 |
| Ro-49 | Romania | Bologa | 46.89084°N 22.88878°E | 2023.04.02 | main road | 506 |
| Ro-50 | Romania | Poieni | 46.93130°N 22.85265°E | 2023.04.02 | main road | 480 |
| Ro-51 | Romania | Ciucea | 46.95850°N 22.80745°E | 2023.04.02 | main road | 461 |
| Ro-52 | Romania | Negreni | 46.96182°N 22.72438°E | 2023.04.02 | main road | 407 |
| Ro-53 | Romania | Bucea | 46.94976°N 22.72079°E | 2023.04.02 | main road | 405 |
| Ro-54 | Romania | Bucea | 46.96247°N 22.68012°E | 2023.04.02 | main road | 487 |
| Ro-55 | Romania | Corniţel | 46.98951°N 22.63073°E | 2023.04.02 | main road | 344 |
| Ro-56 | Romania | Borozel | 47.00001°N 22.56649°E | 2023.04.02 | main road | 297 |
| Ro-57 | Romania | Gheghie | 47.02457°N 22.50874°E | 2023.04.02 | main road | 257 |
| Ro-58 | Romania | Groşi | 47.04911°N 22.46182°E | 2023.04.02 | main road | 243 |
| Ro-59 | Romania | Aleșd | 47.05938°N 22.38074°E | 2023.04.02 | main road | 219 |
| Ro-60 | Romania | Urvind | 47.06493°N 22.31126°E | 2023.04.02 | main road | 205 |
| Ro-61 | Romania | Urvind | 47.07157°N 22.26439°E | 2023.04.02 | main road | 190 |
| Ro-62 | Romania | Tileagd | 47.06641°N 22.17667°E | 2023.04.02 | main road | 166 |
| Ro-63 | Romania | Fughiu | 47.05434°N 22.08557°E | 2023.04.02 | main road | 154 |
| Ro-64 | Romania | Oradea | 47.09764°N 21.92371°E | 2023.04.02 | secondary road | 159 |
| Ro-65 | Romania | Oradea | 47.11334°N 21.89658°E | 2023.04.02 | main road | 113 |
| Ro-66 | Romania | Biharia | 47.17211°N 21.92183°E | 2023.04.02 | main road | 117 |
| Ro-67 | Romania | Satu Nou | 47.19011°N 21.92046°E | 2023.04.02 | main road | 108 |
| Ro-68 | Romania | Tămășeu | 47.22223°N 21.93625°E | 2023.04.02 | main road | 103 |
| Ro-69 | Romania | Mihai Bravu | 47.25662°N 21.92309°E | 2023.04.02 | secondary road | 101 |
| Ro-70 | Romania | Mihai Bravu | 47.26254°N 21.89447°E | 2023.04.02 | secondary road | 100 |
| Ro-71 | Romania | Mihai Bravu | 47.26356°N 21.89057°E | 2023.04.02 | secondary road | 99 |
| Ro-72 | Romania | Roșiori | 47.26176°N 21.96620°E | 2023.04.02 | main road | 121 |
| Ro-73 | Romania | Diosig | 47.29206°N 22.01013°E | 2023.04.02 | main road | 105 |
| Ro-74 | Romania | Nădlac | 46.18492°N 20.77034°E | 2023.04.20 | motorway | 87 |
| Ro-75 | Romania | Nădlac | 46.20985°N 20.79232°E | 2023.04.20 | motorway | 91 |
| Ro-76 | Romania | Nădlac | 46.21010°N 20.79315°E | 2023.04.20 | motorway | 91 |
| Ro-77 | Romania | Nădlac | 46.20785°N 20.78775°E | 2023.04.20 | motorway | 90 |
| Ro-78 | Romania | Pecica | 46.19474°N 21.08924°E | 2023.04.20 | motorway | 102 |
| Ro-80 | Romania | Pecica | 46.17888°N 21.11767°E | 2023.04.20 | motorway | 98 |
| Ro-81 | Romania | Pecica | 46.17887°N 21.11830°E | 2023.04.20 | motorway | 98 |
| Ro-82 | Romania | Arad | 46.16934°N 21.28162°E | 2023.04.20 | motorway | 109 |
| Ro-83 | Romania | Arad | 46.14044°N 21.30680°E | 2023.04.20 | motorway | 114 |
| Ro-84 | Romania | Arad | 46.14001°N 21.30629°E | 2023.04.20 | motorway | 113 |
| Ro-85 | Romania | Arad | 46.20084°N 21.23176°E | 2023.04.20 | motorway | 106 |
| Ro-86 | Romania | Arad | 46.20317°N 21.21240°E | 2023.04.20 | main road | 101 |
| Ro-87 | Romania | Sederhat | 46.23049°N 21.17427°E | 2023.04.20 | main road | 99 |
| Ro-88 | Romania | Turnu | 46.24923°N 21.13617°E | 2023.04.20 | main road | 96 |
| Ro-89 | Romania | Variaşu Mic | 46.29367°N 21.18170°E | 2023.04.20 | secondary road | 99 |
| Ro-90 | Romania | Iratoșu | 46.32172°N 21.21424°E | 2023.04.20 | secondary road | 98 |
| Ro-91 | Romania | Iratoșu | 46.36121°N 21.24720°E | 2023.04.20 | secondary road | 99 |
| Ro-92 | Romania | Comuna Macea | 46.39189°N 21.31929°E | 2023.04.20 | secondary road | 95 |
| Ro-93 | Romania | Socodor | 46.50079°N 21.43378°E | 2023.04.20 | main road | 92 |
| Ro-94 | Romania | Socodor | 46.50262°N 21.44752°E | 2023.04.20 | main road | 89 |
| Ro-95 | Romania | Chișineu-Criș | 46.51103°N 21.48253°E | 2023.04.20 | main road | 89 |
| Ro-96 | Romania | Chișineu-Criș | 46.53668°N 21.52886°E | 2023.04.20 | main road | 90 |
| Ro-97 | Romania | Comuna Ciumeghiu | 46.73994°N 21.58243°E | 2023.04.20 | main road | 90 |
| Ro-98 | Romania | Salonta | 46.78837°N 21.59761°E | 2023.04.20 | secondary road | 90 |
| Ro-99 | Romania | Borș | 47.11525°N 21.80528°E | 2025.04.06 | main road | 105 |
| Srb-01 | Serbia | Horgoš | 46.16613°N 19.95989°E | 2023.04.12 | motorway | 80 |
| Srb-02 | Serbia | Horgoš | 46.14126°N 19.90596°E | 2023.04.12 | motorway | 85 |
| Srb-03 | Serbia | Šupljak | 46.07978°N 19.78453°E | 2023.04.12 | motorway | 102 |
| Srb-04 | Serbia | Bikovo | 45.99851°N 19.72157°E | 2023.04.12 | motorway | 101 |
| Srb-05 | Serbia | Novi Žednik | 45.96662°N 19.71055°E | 2023.04.12 | motorway | 100 |
| Srb-06 | Serbia | Čantavir | 45.88789°N 19.69904°E | 2023.04.12 | motorway | 101 |
| Srb-07 | Serbia | Bačka Topola | 45.79342°N 19.69370°E | 2023.04.12 | motorway | 102 |
| Srb-08 | Serbia | Feketić | 45.67614°N 19.72812°E | 2023.04.12 | motorway | 92 |
| Srb-09 | Serbia | Feketić | 45.62648°N 19.70626°E | 2023.04.12 | motorway | 86 |
| Srb-10 | Serbia | Srbobran | 45.50930°N 19.72484°E | 2023.04.12 | motorway | 77 |
| Srb-11 | Serbia | Zmajevo | 45.45375°N 19.73439°E | 2023.04.12 | motorway | 77 |
| Srb-12 | Serbia | Zmajevo | 45.41334°N 19.73649°E | 2023.04.12 | motorway | 78 |
| Srb-13 | Serbia | Каћ | 45.33006°N 19.92059°E | 2023.04.12 | secondary road | 78 |
| Srb-14 | Serbia | Каћ | 45.27630°N 19.92059°E | 2023.04.12 | motorway | 75 |
| Srb-15 | Serbia | Kovilj | 45.21736°N 20.06266°E | 2023.04.12 | motorway | 77 |
| Srb-16 | Serbia | Beška | 45.15097°N 20.08264°E | 2023.04.12 | motorway | 139 |
| Srb-17 | Serbia | Stara Pazova | 44.99584°N 20.20722°E | 2023.04.12 | motorway | 82 |
| Srb-18 | Serbia | Beograd | 44.73170°N 20.33524°E | 2023.04.12 | motorway | 72 |
| Srb-19 | Serbia | Beograd | 44.71310°N 20.45824°E | 2023.04.12 | secondary road | 167 |
| Srb-20 | Serbia | Mislođin | 44.65050°N 20.23688°E | 2023.04.12 | motorway | 74 |
| Srb-21 | Serbia | Surčin | 44.81097°N 20.25402°E | 2023.04.12 | motorway | 87 |
| Srb-22 | Serbia | Krnješevci | 44.87827°N 20.12264°E | 2023.04.12 | motorway | 75 |
| Srb-23 | Serbia | Šimanovci | 44.88269°N 20.11350°E | 2023.04.12 | motorway | 73 |
| Srb-24 | Serbia | Pećinci | 44.91020°N 19.98896°E | 2023.04.12 | motorway | 78 |
| Srb-25 | Serbia | Dobrinci | 44.93381°N 19.91604°E | 2023.04.12 | motorway | 81 |
| Srb-27 | Serbia | Ruma | 44.94731°N 19.85556°E | 2023.04.12 | motorway | 83 |
| Srb-28 | Serbia | Šašinci | 44.97623°N 19.74549°E | 2023.04.12 | motorway | 89 |
| Srb-29 | Serbia | Sremska Mitrovica | 45.00195°N 19.62295°E | 2023.04.12 | motorway | 83 |
| Srb-30 | Serbia | Laćarak | 45.01855°N 19.53209°E | 2023.04.12 | motorway | 80 |
| Srb-31 | Serbia | Martinc | 45.03573°N 19.45073°E | 2023.04.12 | motorway | 81 |
| Srb-32 | Serbia | Kuzmin | 45.04086°N 19.36809°E | 2023.04.12 | motorway | 77 |
| Srb-33 | Serbia | Adaševci | 45.04170°N 19.25952°E | 2023.04.12 | motorway | 79 |
| Srb-34 | Serbia | Adaševci | 45.04738°N 19.22346°E | 2023.04.12 | motorway | 77 |
| Srb-35 | Serbia | Kuzmin | 45.04069°N 19.36366°E | 2023.04.13 | motorway | 76 |
| Srb-36 | Serbia | Martinci | 45.03522°N 19.45160°E | 2023.04.13 | motorway | 81 |
| Srb-38 | Serbia | Sremska Mitrovica | 45.00150°N 19.62223°E | 2023.04.13 | motorway | 83 |
| Srb-39 | Serbia | Sremska Mitrovica | 44.99939°N 19.63943°E | 2023.04.13 | motorway | 80 |
| Srb-40 | Serbia | Šašinci | 44.97923°N 19.73078°E | 2023.04.13 | motorway | 87 |
| Srb-41 | Serbia | Ruma | 44.95868°N 19.82099°E | 2023.04.13 | motorway | 88 |
| Srb-42 | Serbia | Pećinci | 44.92030°N 19.96037°E | 2023.04.13 | motorway | 79 |
| Srb-43 | Serbia | Šimanovci | 44.87987°N 20.11855°E | 2023.04.13 | motorway | 75 |
| Srb-44 | Serbia | Ugrinovci | 44.85044°N 20.18603°E | 2023.04.13 | motorway | 76 |
| Srb-46 | Serbia | Stari Banovci | 44.97032°N 20.23457°E | 2023.04.13 | motorway | 80 |
| Srb-47 | Serbia | Beška | 45.14554°N 20.08446°E | 2023.04.13 | motorway | 127 |
| Srb-48 | Serbia | Каћ | 45.27586°N 19.92411°E | 2023.04.13 | motorway | 72 |
| Srb-49 | Serbia | Rumenka | 45.32753°N 19.78187°E | 2023.04.13 | motorway | 79 |
| Srb-50 | Serbia | Bačka Topola | 45.79416°N 19.69395°E | 2023.04.13 | motorway | 102 |
| Sk-001 | Slovakia | Milhosť | 48.53269ºN 21.25487ºE | 2023.04.17 | main road | 179 |
| Sk-002 | Slovakia | Seňa | 48.57632ºN 21.25822ºE | 2023.04.17 | main road | 180 |
| Sk-003 | Slovakia | Haniska | 48.62228ºN 21.26540ºE | 2023.04.17 | main road | 194 |
| Sk-004 | Slovakia | Barca | 48.66726ºN 21.27209ºE | 2023.04.17 | main road | 194 |
| Sk-005 | Slovakia | Košice-Ťahanovce | 48.75903ºN 21.28329ºE | 2023.04.17 | main road | 266 |
| Sk-006 | Slovakia | Drienov | 48.85811ºN 21.27008ºE | 2023.04.17 | motorway | 210 |
| Sk-007 | Slovakia | Lemešany | 48.85589ºN 21.27143ºE | 2023.04.17 | motorway | 211 |
| Sk-008 | Slovakia | Petrovany | 48.91435ºN 21.25235ºE | 2023.04.17 | motorway | 220 |
| Sk-009 | Slovakia | Záborské | 48.95109ºN 21.25862ºE | 2023.04.17 | secondary road | 267 |
| Sk-010 | Slovakia | Malý Šariš | 49.00030ºN 21.18752ºE | 2023.04.17 | motorway | 312 |
| Sk-011 | Slovakia | Svinia | 49.00257ºN 21.10435ºE | 2023.04.17 | main road | 351 |
| Sk-012 | Slovakia | Chminianska Nová Ves | 49.01331ºN 21.04844ºE | 2023.04.17 | main road | 390 |
| Sk-013 | Slovakia | Hendrichovce | 49.02147ºN 20.99022ºE | 2023.04.17 | main road | 433 |
| Sk-014 | Slovakia | Široké | 49.00965ºN 20.94319ºE | 2023.04.17 | motorway | 492 |
| Sk-015 | Slovakia | Behárovce | 49.00667ºN 20.81080ºE | 2023.04.17 | main road | 493 |
| Sk-016 | Slovakia | Spišské Podhradie | 49.00730ºN 20.70511ºE | 2023.04.17 | motorway | 471 |
| Sk-017 | Slovakia | Levoča | 49.01128ºN 20.60226ºE | 2023.04.17 | motorway | 564 |
| Sk-018 | Slovakia | Spišský Štvrtok | 49.01455ºN 20.45305ºE | 2023.04.17 | motorway | 568 |
| Sk-019 | Slovakia | Svit | 49.06449ºN 20.18367ºE | 2023.04.17 | motorway | 750 |
| Sk-020 | Slovakia | Štrba | 49.06529ºN 20.08730ºE | 2023.04.17 | motorway | 840 |
| Sk-021 | Slovakia | Štrba | 49.06642ºN 20.06356ºE | 2023.04.17 | motorway | 894 |
| Sk-022 | Slovakia | Východná | 49.07104ºN 19.91488ºE | 2023.04.17 | motorway | 874 |
| Sk-023 | Slovakia | Hybe | 49.05792ºN 19.82856ºE | 2023.04.17 | motorway | 742 |
| Sk-024 | Slovakia | Liptovský Mikuláš | 49.07738ºN 19.60017ºE | 2023.04.17 | secondary road | 589 |
| Sk-025 | Slovakia | Galovany | 49.09205ºN 19.51545ºE | 2023.04.17 | motorway | 569 |
| Sk-026 | Slovakia | Ivachnová | 49.09081ºN 19.40625ºE | 2023.04.17 | main road | 502 |
| Sk-027 | Slovakia | Ružomberok | 49.09663ºN 19.25523ºE | 2023.04.17 | main road | 463 |
| Sk-028 | Slovakia | Hubová | 49.12175ºN 19.19711ºE | 2023.04.17 | main road | 446 |
| Sk-029 | Slovakia | Stankovany | 49.14484ºN 19.16682ºE | 2023.04.17 | main road | 436 |
| Sk-030 | Slovakia | Štiavnička | 49.09998ºN 19.03066ºE | 2023.04.17 | motorway | 422 |
| Sk-031 | Slovakia | Bytča | 49.21546ºN 18.55559ºE | 2023.04.18 | secondary road | 300 |
| Sk-032 | Slovakia | Predmier | 49.20286ºN 18.52241ºE | 2023.04.18 | motorway | 297 |
| Sk-033 | Slovakia | Beluša | 49.08189ºN 18.33029ºE | 2023.04.18 | motorway | 259 |
| Sk-034 | Slovakia | Považská Bystrica | 49.11535ºN 18.43492ºE | 2023.04.18 | motorway | 325 |
| Sk-035 | Slovakia | Púchov | 49.12342ºN 18.30869ºE | 2023.04.18 | main road | 274 |
| Sk-036 | Slovakia | Dohňany | 49.16223ºN 18.27498ºE | 2023.04.18 | main road | 301 |
| Sk-037 | Slovakia | Lúky | 49.18692ºN 18.23899ºE | 2023.04.18 | main road | 334 |
| Sk-038 | Slovakia | Lysá pod Makytou | 49.19356ºN 18.16472ºE | 2023.04.18 | main road | 402 |
| Sk-039 | Slovakia | Horné Srnie | 49.02593ºN 18.07118ºE | 2023.04.18 | main road | 282 |
| Sk-040 | Slovakia | Horné Srnie | 48.99102ºN 18.10548ºE | 2023.04.18 | main road | 245 |
| Sk-041 | Slovakia | Nemšová | 48.96572ºN 18.13153ºE | 2023.04.18 | main road | 222 |
| Sk-042 | Slovakia | Zamarovce | 48.91356ºN 18.05650ºE | 2023.04.18 | motorway | 263 |
| Sk-043 | Slovakia | Trenčín | 48.90116ºN 17.99684ºE | 2023.04.18 | main road | 207 |
| Sk-044 | Slovakia | Beckov | 48.80078ºN 17.89587ºE | 2023.04.18 | motorway | 188 |
| Sk-045 | Slovakia | Beckov | 48.76272ºN 17.86782ºE | 2023.04.18 | motorway | 183 |
| Sk-046 | Slovakia | Považany | 48.70889ºN 17.87218ºE | 2023.04.18 | motorway | 173 |
| Sk-047 | Slovakia | Horná Streda | 48.64936ºN 17.84122ºE | 2023.04.18 | motorway | 169 |
| Sk-048 | Slovakia | Žlkovce | 48.45381ºN 17.73877ºE | 2023.04.18 | motorway | 145 |
| Sk-049 | Slovakia | Zeleneč | 48.32675ºN 17.61054ºE | 2023.04.18 | motorway | 137 |
| Sk-050 | Slovakia | Zeleneč | 48.30885ºN 17.56120ºE | 2023.04.18 | motorway | 148 |
| Sk-051 | Slovakia | Igram | 48.27506ºN 17.46526ºE | 2023.04.18 | motorway | 136 |
| Sk-052 | Slovakia | Senec | 48.24422ºN 17.38923ºE | 2023.04.18 | motorway | 135 |
| Sk-053 | Slovakia | Bratislava-Ružinov | 48.18718ºN 17.20395ºE | 2023.04.18 | motorway | 133 |
| Sk-054 | Slovakia | Záhorská Bystrica | 48.20838ºN 17.03831ºE | 2023.04.18 | motorway | 181 |
| Sk-055 | Slovakia | Stupava | 48.25991ºN 17.01019ºE | 2023.04.18 | motorway | 164 |
| Sk-056 | Slovakia | Lozorno | 48.32517ºN 17.02969ºE | 2023.04.18 | secondary road | 173 |
| Sk-057 | Slovakia | Lozorno | 48.32512ºN 17.02891ºE | 2023.04.18 | main road | 173 |
| Sk-058 | Slovakia | Lozorno | 48.32437ºN 17.02369ºE | 2023.04.18 | motorway | 175 |
| Sk-059 | Slovakia | Lozorno | 48.32976ºN 17.03227ºE | 2023.04.18 | secondary road | 176 |
| Sk-060 | Slovakia | Lozorno | 48.33572ºN 17.05633ºE | 2023.04.18 | secondary road | 199 |
| Sk-061 | Slovakia | Lozorno | 48.31481ºN 17.02864ºE | 2023.04.18 | main road | 179 |
| Sk-062 | Slovakia | Lozorno | 48.32874ºN 17.01844ºE | 2023.04.18 | main road | 165 |
| Sk-063 | Slovakia | Lozorno | 48.33424ºN 17.01559ºE | 2023.04.18 | main road | 164 |
| Sk-064 | Slovakia | Malacky | 48.42867ºN 17.03895ºE | 2023.04.18 | secondary road | 171 |
| Sk-065 | Slovakia | Sekule | 48.61395ºN 17.01325ºE | 2023.04.18 | motorway | 164 |
| Sk-066 | Slovakia | Kúty | 48.64392ºN 17.00720ºE | 2023.04.18 | main road | 151 |
| Sk-067 | Slovakia | Kúty | 48.63865ºN 17.00090ºE | 2023.04.18 | main road | 152 |
| Sk-068 | Slovakia | Kúty | 48.63832ºN 17.00195ºE | 2023.04.18 | motorway | 153 |
| Sk-069 | Slovakia | Kúty | 48.63862ºN 17.00226ºE | 2023.04.18 | motorway | 153 |
| Sk-070 | Slovakia | Brodské | 48.68126ºN 16.98718ºE | 2023.04.19 | motorway | 156 |
| Sk-071 | Slovakia | Sekule | 48.61690ºN 17.01203ºE | 2023.04.19 | motorway | 163 |
| Sk-072 | Slovakia | Malacky | 48.46342ºN 17.03645ºE | 2023.04.19 | motorway | 163 |
| Sk-073 | Slovakia | Stupava | 48.26261ºN 17.00936ºE | 2023.04.19 | motorway | 165 |
| Sk-074 | Slovakia | Jarovce | 48.08791ºN 17.09878ºE | 2023.04.19 | motorway | 136 |
| Sk-075 | Slovakia | Rusovce | 48.06840ºN 17.14251ºE | 2023.04.19 | main road | 132 |
| Sk-076 | Slovakia | Podunajské Biskupice | 48.09349ºN 17.21007ºE | 2023.04.19 | motorway | 129 |
| Sk-077 | Slovakia | Ivanka pri Dunaji | 48.19397ºN 17.24104ºE | 2023.04.19 | main road | 131 |
| Sk-078 | Slovakia | Senec | 48.24391ºN 17.38927ºE | 2023.04.19 | motorway | 137 |
| Sk-079 | Slovakia | Čataj | 48.27224ºN 17.46263ºE | 2023.04.19 | motorway | 132 |
| Sk-080 | Slovakia | Zeleneč | 48.32453ºN 17.60655ºE | 2023.04.19 | motorway | 133 |
| Sk-081 | Slovakia | Dolná Streda | 48.25861ºN 17.75609ºE | 2023.04.19 | motorway | 122 |
| Sk-082 | Slovakia | Báb | 48.29663ºN 17.89432ºE | 2023.04.19 | motorway | 198 |
| Sk-083 | Slovakia | Veľké Zálužie | 48.32482ºN 17.94451ºE | 2023.04.19 | motorway | 186 |
| Sk-084 | Slovakia | Pohranice | 48.31664ºN 18.16457ºE | 2023.04.19 | motorway | 213 |
| Sk-085 | Slovakia | Čaradice | 48.35517ºN 18.49649ºE | 2023.04.19 | main road | 264 |
| Sk-086 | Slovakia | Čaradice | 48.35687ºN 18.49535ºE | 2023.04.19 | motorway | 275 |
| Sk-087 | Slovakia | Tekovské Nemce | 48.37031ºN 18.53435ºE | 2023.04.19 | motorway / parking place | 303 |
| Sk-088 | Slovakia | Tekovské Nemce | 48.37140ºN 18.53158ºE | 2023.04.19 | motorway | 312 |
| Sk-089 | Slovakia | Voznica | 48.46198ºN 18.69093ºE | 2023.04.19 | motorway | 230 |
| Sk-090 | Slovakia | Lehôtka pod Brehmi | 48.54808ºN 18.81733ºE | 2023.04.19 | main road | 246 |
| Sk-091 | Slovakia | Lehôtka pod Brehmi | 48.54937ºN 18.81500ºE | 2023.04.19 | motorway | 234 |
| Sk-092 | Slovakia | Budča | 48.56815ºN 19.05937ºE | 2023.04.19 | motorway | 271 |
| Sk-093 | Slovakia | Zvolen | 48.56028ºN 19.08432ºE | 2023.04.19 | motorway | 295 |
| Sk-094 | Slovakia | Zvolen | 48.57091ºN 19.18155ºE | 2023.04.19 | main road | 314 |
| Sk-095 | Slovakia | Vígľaš | 48.56305ºN 19.31264ºE | 2023.04.19 | motorway | 345 |
| Sk-096 | Slovakia | Stožok | 48.54228ºN 19.34870ºE | 2023.04.19 | motorway | 354 |
| Sk-097 | Slovakia | Detva | 48.53328ºN 19.41342ºE | 2023.04.19 | motorway | 377 |
| Sk-098 | Slovakia | Kriváň | 48.53017ºN 19.45504ºE | 2023.04.19 | secondary road | 398 |
| Sk-099 | Slovakia | Píla | 48.48437ºN 19.51556ºE | 2023.04.19 | main road | 299 |
| Sk-100 | Slovakia | Mýtna | 48.46281ºN 19.56572ºE | 2023.04.19 | main road | 250 |

**Table S3.** Annual precipitation totals (mm) at three meteorological stations representing the surveyed continental roadside sites in Hungary and at six reference localities across the native Atlantic coastal range of Cochlearia danica during 2004–2023. Summary statistics are provided as the mean, median, standard deviation (SD), minimum and maximum of the annual precipitation totals. Hungarian station data were obtained from HungaroMet Nonprofit Zrt. (2024), whereas precipitation values for the Atlantic reference localities were derived from the E-OBS gridded observational dataset (Cornes et al. 2018).

|  | **Hungary** | **Hungary** | **Hungary** |  |  |  |
| --- | --- | --- | --- | --- | --- | --- |
| **Year** | **Túrkeve** | **Debrecen** | **Szeged** |  |  |  |
| 2004 | 733,4 | 694,3 | 688,5 |  |  |  |
| 2005 | 785,9 | 644,9 | 652,6 |  |  |  |
| 2006 | 588,6 | 633 | 501,9 |  |  |  |
| 2007 | 563,6 | 550,7 | 556 |  |  |  |
| 2008 | 507,5 | 551,1 | 527,2 |  |  |  |
| 2009 | 617,9 | 484,6 | 510,3 |  |  |  |
| 2010 | 796,5 | 844,8 | 837,6 |  |  |  |
| 2011 | 363,6 | 441,6 | 305,2 |  |  |  |
| 2012 | 401,7 | 427 | 401,5 |  |  |  |
| 2013 | 544,9 | 544 | 545,2 |  |  |  |
| 2014 | 782,8 | 455,5 | 841,3 |  |  |  |
| 2015 | 459,8 | 432 | 441,6 |  |  |  |
| 2016 | 664,5 | 743,9 | 641,6 |  |  |  |
| 2017 | 571,8 | 569,1 | 437,7 |  |  |  |
| 2018 | 473,7 | 434,7 | 566,7 |  |  |  |
| 2019 | 581,9 | 455,8 | 510,8 |  |  |  |
| 2020 | 624,9 | 571,4 | 554,7 |  |  |  |
| 2021 | 382,3 | 379,9 | 454,6 |  |  |  |
| 2022 | 339,4 | 441 | 385,1 |  |  |  |
| 2023 | 584,4 | 663,5 | 515,3 |  |  |  |
|  |  |  |  |  |  |  |
| **Mean** | 568 | 548 | 544 |  |  |  |
| **Median** | 577 | 547 | 521 |  |  |  |
| **SD** | 140 | 124 | 137 |  |  |  |
| **Min** | 339 | 380 | 305 |  |  |  |
| **Max** | 797 | 845 | 841 |  |  |  |
|  |  |  |  |  |  |  |
|  | **Portugal** | **UK** | **Ireland** | **the Netherlands** | **France** | **Denmark** |
| **Year** | **Venade** | **Fishguard** | **Ferrypoint** | **Midelburg** | **Lampaul-Ploudalmézeau** | **Øsløs** |
| 2004 | 952,6 | 862,8 | 845,8 | 697,4 | 761,6 | 741,2 |
| 2005 | 996,6 | 743,9 | 778,8 | 837,8 | 733,5 | 645,1 |
| 2006 | 1818,9 | 886,5 | 1084,7 | 642,5 | 831 | 954,1 |
| 2007 | 980,8 | 952,6 | 948,3 | 833,5 | 882,9 | 740,6 |
| 2008 | 1387,1 | 1181,2 | 1082 | 768,4 | 949,5 | 816,2 |
| 2009 | 1546,5 | 1071,5 | 1348,9 | 653,7 | 955,5 | 781,2 |
| 2010 | 1501,3 | 784,4 | 749,3 | 819,9 | 906 | 605,6 |
| 2011 | 1087 | 927,5 | 722,7 | 765,5 | 658 | 714,8 |
| 2012 | 1158,9 | 1325,5 | 839,1 | 951,2 | 1122,9 | 781,1 |
| 2013 | 1746,9 | 1190,9 | 899,1 | 850,4 | 878,7 | 737,2 |
| 2014 | 1982,7 | 1334,1 | 1007,5 | 785,2 | 1097,6 | 812,5 |
| 2015 | 1253,4 | 1424,6 | 979,4 | 781,1 | 873,6 | 896,4 |
| 2016 | 1622,2 | 1224,6 | 877,8 | 802,3 | 795,2 | 591 |
| 2017 | 1120,9 | 1348,7 | 916,7 | 934,3 | 710,6 | 722 |
| 2018 | 1625,2 | 1351,6 | 979,9 | 676,3 | 807,7 | 568,2 |
| 2019 | 1332,9 | 1453 | 951,6 | 678,5 | 951,3 | 961 |
| 2020 | 1407,6 | 1059,3 | 1015 | 803,3 | 965,3 | 762,8 |
| 2021 | 1272,3 | 903,9 | 859,9 | 822,3 | 787,7 | 643,4 |
| 2022 | 1619,6 | 744,1 | 811,7 | 702,7 | 714,3 | 696,8 |
| 2023 | 1753,9 | 1209,6 | 1086,8 | 941 | 825,9 | 829,7 |
|  |  |  |  |  |  |  |
| **Mean** | 1408 | 1099 | 939 | 787 | 860 | 750 |
| **Median** | 1397 | 1126 | 933 | 794 | 852 | 741 |
| **SD** | 304 | 235 | 145 | 93 | 123 | 110 |
| **Min** | 953 | 744 | 723 | 643 | 658 | 568 |
| **Max** | 1983 | 1453 | 1349 | 951 | 1123 | 961 |

**Fig. S1.** Documented roadside populations of *Cochlearia danica* newly recorded in Romania and Serbia. Photographs show populations at Pecica (Romania) (A), Ruma (Serbia) (B), and Laćarak (Serbia) (C), illustrating local establishment in regions where the species had not previously been reported.

**Fig. S2.** Changes in population size of selected *Cochlearia danica* populations between survey years. The figure compares population estimates from 2023–2024 with resurvey data from 2025, indicating persistence and local population growth at several sites. Please note that the scale of each Y axis is different and comparisons between populations should be taken accordingly.

**Fig. S3.** Geographic distribution of surveyed roadside sampling points and known occurrences of Cochlearia danica in the Pannonian Basin and adjacent regions. Surveyed sampling points with C. danica (red circles) and without C. danica (open circles) are shown, together with previously published occurrence records (black circles) from Fekete et al. (2018), Schmidt et al. (2023), Süveges (2025) and Takács et al. (2025).
